# Personalized high-intensity temporal interference stimulation decouples cerebellar networks to enhance implicit learning

**DOI:** 10.1186/s12984-025-01865-9

**Published:** 2026-01-02

**Authors:** Dongsheng Tang, Lang Qin, Longfei Hu, Yixuan Jian, Siqi Gao, Zhenhe Huang, Liangling Cai, Xueyun Shao, Chunqi Chang, Gang Zhao, Zhiqiang Zhu

**Affiliations:** 1 School of Kinesiology, Normal College, Shenzhen University, Room B-428, Nanhai Avenue, Shenzhen, 518060 Guangdong China; 2 Department of Geriatrics, Affiliated Nanshan Hospital of Shenzhen University, Shenzhen, 518060 China; 3 School of Biomedical Engineering, Shenzhen University Medical School, Shenzhen University, Shenzhen, 518060 China; 4 Magnetic Resonance Imaging (MRI) Center, Shenzhen University, Shenzhen, 518060 China

**Keywords:** Temporal interference stimulation, Noninvasive brain stimulation, Cerebellar network, Structure-function couplings, Implicit learning, Network connection

## Abstract

**Objective:**

Despite the central role of deep brain structures such as the striatum in motor learning, existing noninvasive stimulation methods are hindered by limited depth and precision. Temporal interference (TI) stimulation presents the potential for precise, individualized modulation of deep regions. However, how TI stimulation influences deep brain activity and large-scale network reorganization to facilitate motor learning is still unclear. Therefore, this study aimed to clarify these mechanisms by investigating how personalized, high-intensity TI targeting the striatum modulates neural activity and enhances motor learning.

**Methods:**

Twenty-six healthy right-handed male participants were enrolled in a randomized, double-blind, sham-controlled crossover study. Each participant received both TI and sham stimulation targeting the right striatum (10 mA, Δf = 20 Hz) through an individualized electrode montage. Resting-state functional magnetic resonance imaging (fMRI), diffusion tensor imaging (DTI), and serial reaction time (SRT) task performance were assessed before and after each intervention. Neural analyses included static and dynamic fractional amplitude of low-frequency fluctuation (fALFF) in the target region, structural connectivity (SC)–functional connectivity (FC) coupling and topological metrics across six major brain networks, as well as brain-behavior correlations related to learning performance.

**Results:**

(1) Target region activity: TI stimulation significantly increased both static and dynamic fALFF in the right striatum (Cohen’s d = 0.49, *p* = 0.016; Cohen’s d = 0.39, *p* = 0.035). (2) Brain network reorganization: Compared with the Sham group, the TI group exhibited significantly reduced SC-FC coupling in the cerebellar network (CN) (t = – 2.279, Cohen’s d = -0.82, *p* = 0.027, FDR corrected). TI stimulation significantly enhanced nodal degree in the cerebellar network (CN) and nodal efficiency the cingulo-opercular network (CON) (FDR corrected with Bonferroni correction, *p* < 0.0083) following the stimulation. Behavioral performance: The TI stimulation significantly improved performance in second implicit learning (SIL) after stimulation (Cohen’s d = 0.47, *p* = 0.043). Brain-behavior correlation: Changes in SC-FC coupling in the CN were significantly negatively correlated with improvement in SIL (*r* = – 0.372, *p* = 0.040).

**Conclusion:**

Personalized high-intensity TI of the striatum enhances deep target activity and promotes selective network reorganization, particularly by reducing SC-FC coupling and strengthening intra-network connectivity in the CN. These network-level modulations underlie improved implicit learning performance, highlighting the potential of TI neuromodulation as a precise and effective approach for promoting motor learning by targeting deep nuclei and large-scale brain networks.

*Trial registration number* ChiCTR2500098699.

**Supplementary Information:**

The online version contains supplementary material available at 10.1186/s12984-025-01865-9.

## Introduction

The striatum, as a core node of the basal ganglia, plays a pivotal role in complex cognitive functions such as motor learning and control mechanisms [1, 2]. In recent years, increasing evidence suggests that the striatum plays a crucial role in implicit learning, especially in unconscious skill acquisition and behavioral adaptation [3, 4]. Precise regulation of striatal function shows immense application potential in fields such as brain function research, neuropsychiatric intervention, and cognitive enhancement [5, 6].

However, due to the striatum’s deep brain structural location, traditional non-invasive stimulation techniques such as transcranial direct current stimulation (tDCS) and transcranial magnetic stimulation (TMS) have significant limitations in terms of stimulation depth and localization accuracy [7, 8]. Temporal Interference (TI) Stimulation, by forming a low-frequency interference field within brain tissue through two high-frequency currents, achieves precise, non-invasive regulation of deep neural structures [9]. TI stimulation not only avoids over-activation of superficial tissues, significantly reduces side effects, but also possesses higher spatial resolution, thus being widely considered a powerful tool for non-invasive deep brain modulation [10, 11].

The efficiency and safety of TI stimulation have been initially verified. Yang et al. demonstrated that TI stimulation of the globus pallidus interna significantly improved motor function in patients with Parkinson’s disease (PD) [12]. Similarly, Lamoš et al. indicated that stimulation of the subthalamic nucleus in Parkinson’s patients could reverse abnormal brain discharges [13]. However, differences in stimulation paradigms (including stimulation parameters and electrode configuration) significantly affect the efficacy of neuromodulation. Wessel et al. found that 2 mA TI stimulation of the striatum significantly increased blood oxygen level-dependent (BOLD) signal activation under task-based functional magnetic resonance imaging (fMRI), yet no significant changes in functional connectivity were observed during resting-state fMRI [6]. No significant differences were observed in resting-state functional imaging, which may be due to insufficient electric field strength in the target region. Because the currents dissipates with increasing depth, it may fail to generate sufficient field strength to activate the intended targets [14]. Cassarà et al. indicated that for weak applied fields (< 1 V/m) [15, 16], exogenous polarization interacts with ongoing dynamics to bias spike timing through stochastic resonance, while overt network entrainment or rhythm replacement typically requires higher field strengths [16–19]. Therefore, increasing the intensity of TI stimulation may be a key factor in achieving more effective stimulation. Moreover, accumulating evidence suggests that non-invasive transcranial electrical stimulation modulates brain network dynamics and influences implicit learning performance [20]. However, the systematic exploration of how TI regulates striatal network function and the corresponding influences on implicit learning behavior remains poorly understood.

Therefore, this study takes individualized high-intensity TI stimulation as a starting point, optimizing parameters based on individual brain structural differences, and combining multimodal brain imaging and behavioral indicators to systematically evaluate the causal effect of TI on the decoupling of striatum-related brain networks and the improvement of implicit learning performance. The study hypothesizes that personalized high-intensity TI stimulation of the striatum can effectively regulate the functional and structural networks of the brain closely associated with the striatum, and promote individual implicit learning ability, providing new theoretical and methodological bases for non-invasive precise regulation of deep brain regions and cognitive function intervention.

## Methods

### Participants

26 healthy male participants (mean age 20.31 ± 1.69 years) were included in the study. All participants were right-handed (mean laterality quotient 85.90 ± 17.44), had no history of neurological or severe psychiatric diseases, medication use, or metal implants. After quality-control procedures, three participants were excluded from serial reaction time (SRT) tasks analysis owing to explicit awareness of the task repetition. This study approved by the Medical Ethics Committee of Shenzhen University Health Science Center (project number 202400151) and pre-registered on the Chinese Clinical Trial Registry (www.chictr.org.cn; identifier: ChiCTR2500098699). All subjects provided written informed consent and the experimental procedures were performed in accordance with the Declaration of Helsinki.

### Study design

**Fig. 1:**
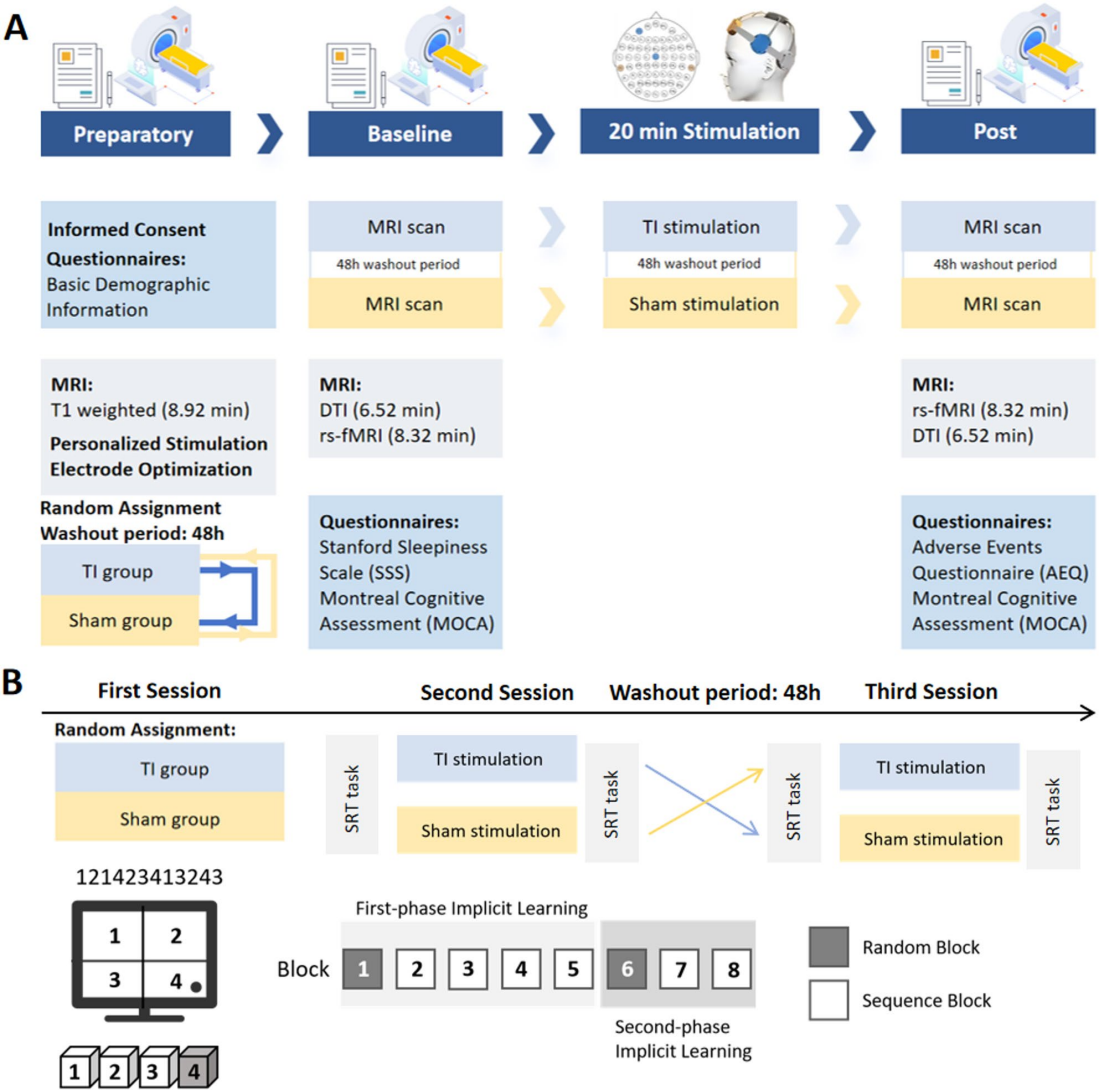
Study design.** A** Magnetic resonance imaging experimental protocol.** B** Serial reaction time task protocol.* TI* temporal interference stimulation,* rs-fMRI* resting-state functional magnetic resonance imaging,* DTI* diffusion tensor imaging,* SRT* serial reaction time task

The study employed a randomized double-blind sham-controlled cross-over design. All participants underwent two experiments: the magnetic resonance imaging (MRI) experiment (Fig. 1A) and the SRT task experiment (Fig. 1B). In the MRI experiment, all participants underwent three experimental sessions. At the first session, demographic data were collected, and high-resolution T1-weighted MRI scans were acquired for personalized stimulation electrode protocol optimization. For the subsequent two sessions, participants were randomly assigned to either the TI group (TI stimulation) or the Sham group (sham stimulation), with a minimum of 48 h washout period between sessions. Resting-state fMRI and diffusion tensor imaging (DTI) data were collected before and after stimulation during each session. Additionally, participants completed the Stanford Sleepiness Scale (SSS) prior to stimulation and the Adverse Events Questionnaire (AEQ) following stimulation. The Montreal Cognitive Assessment (MoCA) was administered both before and after each stimulation session (Fig. 1A).

In the SRT task experiment, participants underwent the same cross-over design across two sessions, with SRT performance assessed before and after each stimulation intervention. The SRT task was performed on laptops using E-Prime V3.0 software (Psychology Software Tools, Inc., Pittsburgh, PA, USA). Participants were instructed to position their non-dominant left hand over the response pad, with the index, middle, ring, and little fingers placed over buttons 4, 3, 2, and 1, respectively. Participants were instructed to respond as rapidly as possible by pressing the corresponding keys with their pre-positioned fingers in response to the numbers indicated by asterisks displayed on the computer screen. The stimuli disappeared immediately after pressing any key and reappeared after a 500 ms interval [21].

SRT task comprised eight blocks, each containing 120 trials. Blocks 1 and 6, termed random (“R”) blocks, presented the sequence of asterisks in a pseudorandom order. For both R blocks, the asterisks appeared with equal frequency at each position, and the sequence was constrained to prevent runs of four consecutive unique positions (e.g., 1234 or 4321) or trills of four elements (e.g., 1212). In contrast, Blocks 2–5 and 7–8, designated as sequence (“S”) blocks, featured a fixed 12-element sequence of asterisk positions (121423413243), which was repeated ten times per block. The task design encompassed two distinct learning phases. Denoting the reaction time of block n as RTn, the first implicit learning (FIL) was defined as FIL = RT1 – (RT2 + RT3 + RT4 + RT5)/4, while the second implicit learning (SIL) was defined as SIL = RT6 – (RT7 + RT8)/2. Prior to the experimental session, participants completed a 60-trial practice block presented in a random order to ensure comprehension of the task instructions. A 30-second rest interval was provided between each block. Importantly, participants were not informed about the presence of a repeating sequence. Upon completion of the experiment, participants were queried as to whether they had noticed any repeated sequences. Those who reported awareness of the sequence were excluded from the final analysis.

### Stimulation parameter and personalized stimulus montage protocol

**Fig. 2:**
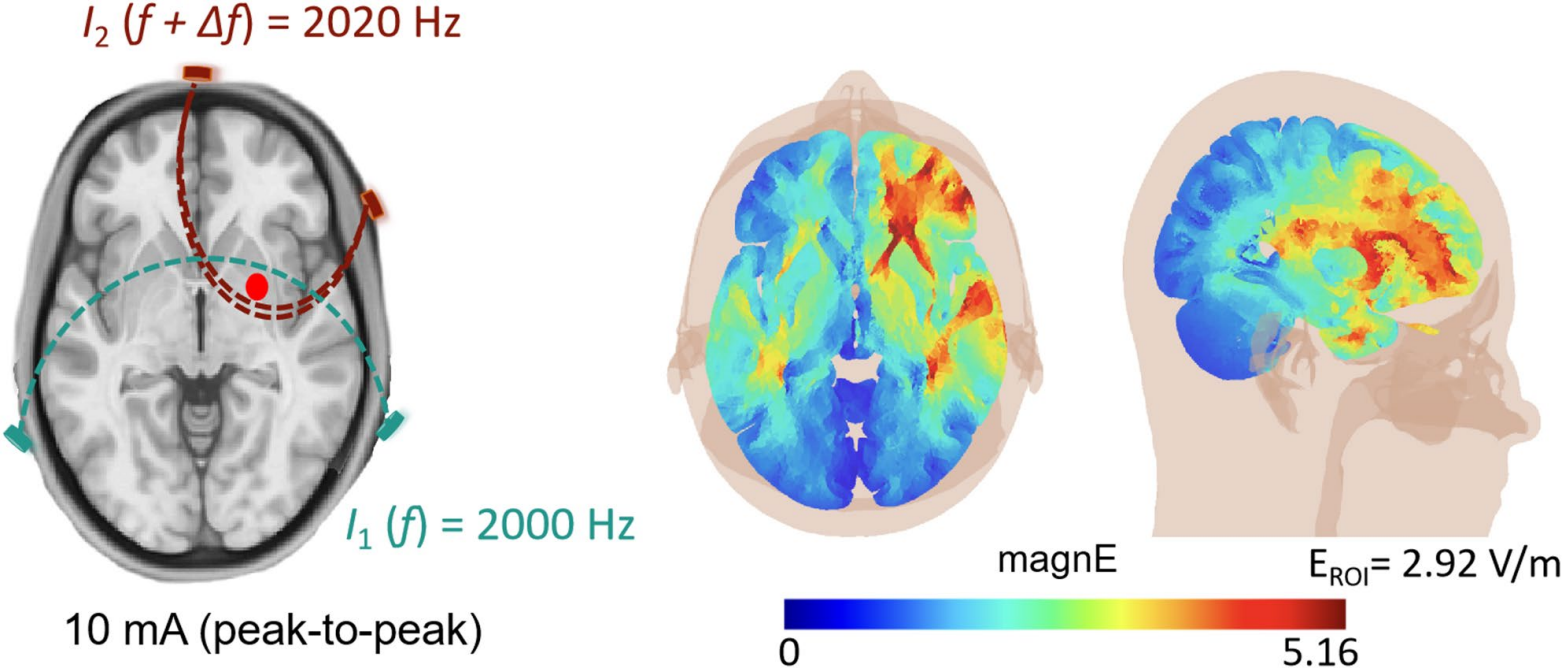
Concept map of electric field modeling with the right striatum. Note: The colors show the temporal interference exposure (electric field modulation magnitude)

The intervention was conducted by the NervioX-1000 neuromodulation system (Suzhou Brain Dome Technology Co., Ltd., Suzhou, China). The stimulation region-of-interest (ROI) was located in the right striatum. Circular rubber electrodes with a radius of 2 cm were securely mounted on a 10–10 EEG cap, and sufficient Signagel Electrode Gel^®^ was applied between the electrodes and the scalp. Two channels of high-frequency alternating current were applied (I₁: 2 kHz and I₂: 2.02 kHz), generating a low frequency interference modulation of 20 Hz targeting the ROI (Fig. 2). The peak-to‐peak amplitude of the current was set at 10 mA. The duration of TI protocol was 20 min. Previous studies confirm the safety of high-intensity stimulation parameters, establishing that 20-minute durations and currents up to 14 mA are well-tolerated without adverse outcomes [12, 22]. The parameters of Sham group protocol were identical to those of TI group protocol, except that the current was only delivered at the first 30 s and the last 30 s of the whole stimulation session. To monitor potential side effects during the high-intensity TI protocol, we utilized Adverse Effects Questionnaires. Participants rated their sensations every 5 min on a 4-point scale (0 = none, 1 = mild, 2 = moderate, 3 = strong). Stimulation was terminated if a participant reported a level 2 sensation or higher, but none did. After stimulation, participants evaluated sensations such as itching, pain, and fatigue, with statistical analysis revealing no significant adverse effects between the TI and Sham groups (χ² = 10.864, *p* = 0.285) (Fig. S4). Detailed information is in *Supplementary Section B.* Additionally, we continuously monitored electrode-skin impedance with a constant-current tester, maintaining values below 2 kΩ to minimize skin sensation and ensure stable current transmission. During concurrent MRI scanning, impedance dropped further to approximately 1.5 kΩ, resulting in a scalp voltage of approximately 15 V, well within safety limits.

The electrode locations were optimized for each participant. This was done using the SimNIBS software to create a finite element model (FEM) of the brain from the structural images of the subject [23]. Specifically, we segmented tissues and assigned conductivities, placed electrodes following the standard 10–10 EEG system of 64 channels, generated tetrahedral head meshes via Gmsh, performed FEM, and then calculated the electric field. The right striatum was targeted at MNI coordinates ( [4, 28], – [4]) from Wessel et al. [6], using a 10-mm spherical ROI to optimize electric field intensity. This approach achieved an average electric field intensity of 2.92 V/m in the target region, ensuring precise neuromodulation (for detailed electrode placement and electromagnetic computation data, see *Supplementary Section A*).

### Image acquisition

All MRI data were acquired at the Center for Magnetic Resonance Imaging, Shenzhen University using a Siemens Prisma 3.0-Tesla system (Erlangen, Germany) equipped with a 64-channel head coil. Rs-fMRI scans were acquired using gradient-echo echo-planar imaging (EPI) sequences with the following parameters: 3 × 3 × 3 mm^3^ voxel size, repetition time (TR) = 1000 ms, echo time (TE) = 30 ms, flip angle (FA) = 66°, field of view (FOV) = 210 × 210 mm^2^, total acquisition time (TA) = 8.32 min, 488 volumes. T1 images were acquired using a 3D MPRAGE (magnetization-prepared rapid gradient echo) sequence with the following parameters: 1 × 1 × 1 mm^3^ voxel size, TR = 2300 ms, TE = 2.26 ms, inverse time = 1000 ms, FA = 8°, FOV = 256 × 232 mm^2^, TA = 8.92 min, 192 volumes. A diffusion-weighted spin-echo echo-planar imaging sequence was acquired with the following parameters: 1.5 × 1.5 × 1.5 mm^3^ voxel size, TR = 5600 ms, TE = 82.0 ms, FOV = 210 × 210 mm^2^, TA = 6.52 min, 66 volumes, phase encoding direction = P to A, 64 directions (b = 1500s/mm²)). Participants wore earplugs for noise protection and were instructed to remain awake, still, and focused on a fixation cross with open eyes, avoiding directed thoughts during the scanning sessions.

### Data preprocessing

The rs-fMRI data were preprocessed using DPARSF V8.0 toolboxes [24]. The first 8 volumes were discarded. Images then underwent slice timing, head motion correction, and spatial normalization to the Montreal Neurological Institute (MNI) space by Diffeomorphic Anatomical Registration Through Exponentiated Lie algebra (DARTEL) [25]. After normalization, the linear trends were removed and the nuisance variables, including Friston 24 head motion parameters, white matter (WM), cerebrospinal fluid signals (CSF) and global signal [26], were regressed out from the functional signal. Finally, band-pass filtering (0.01–0.1 Hz) was performed on the images to reserve low-frequency information. To mitigate head motion effects, volume-based frame-wise displacement (FD) was calculated [27]. Timepoints with FD > 0.2 mm were marked as problematic and included as separate regressors during nuisance covariate regression [28]. Participants with mean FD exceeding three standard deviations were excluded from analysis. Finally, 2 participants were excluded under the head motion control criteria, and 26 participants were included in the subsequent analysis.

DWI data were pre-processed using Mrtrix3 (http://www.mrtrix.org/). With the following operations: denoise, remove Gibbs Ringing artifacts. Using FSL’s eddy tool (https://fsl.fmrib.ox.ac.uk/fsl/) inhomogeneity distortion correction, corrected for eddy currents and motion artifacts [29]. Bias field correction using the N4 algorithm as provided in ANTs (https://github.com/ANTsX/ANTs) [30]. Using dhollander method to estimate response function for spherical deconvolution and multi-shell multi-tissue CSD to estimate fiber orientation distributions from diffusion data using spherical deconvolution. Before performing streamlines tractography, conduct multi-tissue informed log-domain intensity normalization. Performing streamlines tractography using second-order integration over fiber orientation distributions option, 10 million streamlines are to be selected. Finally, tcksift was employed to filter the whole-brain fiber-tracking dataset, ensuring that the streamline densities matched the fiber orientation distribution lobe integrals.

### Calculation of static and dynamic fALFF in target regions

The analysis was conducted using the unfiltered, preprocessed data. A fast Fourier transform was performed on whole-brain voxels to convert the BOLD signal into the frequency-domain power spectrum. The square root of the power spectrum was calculated at each frequency, and the average value within the range of 0.01 to 0.10 Hz was used to calculate the static fALFF metric. The resulting static fALFF maps were further subjected to standardization and spatial smoothing with a full width at half maximum (FWHM) of 4 mm. The analysis of dynamic fALFF was performed using the DPABI-based dynamic analysis toolbox. The Hamming sliding window was selected for the whole-brain blood oxygenation level dependent signal time series :100TR (100s) window length and step width of 3 TR (3s) were selected for dynamic fALFF analysis [31]. The mean dynamic fALFF metric was then calculated across all voxels within the 127 windows for each participant to assess the dynamic characteristics of fALFF. For regions of interest (ROIs), we extracted both static and mean dynamic fALFF values from a 5-mm radius sphere centered at the MNI coordinates [4, 28), corresponding to the right striatum (the target of stimulation). As a control region, we also derived measures from bilateral middle frontal gyrus and middle temporal gyrus, defined based on the Anatomical Automatic Labeling (AAL) template, as well as from the area subjacent to the electrode placement.

### Functional and structural network construction

**Fig. 3:**
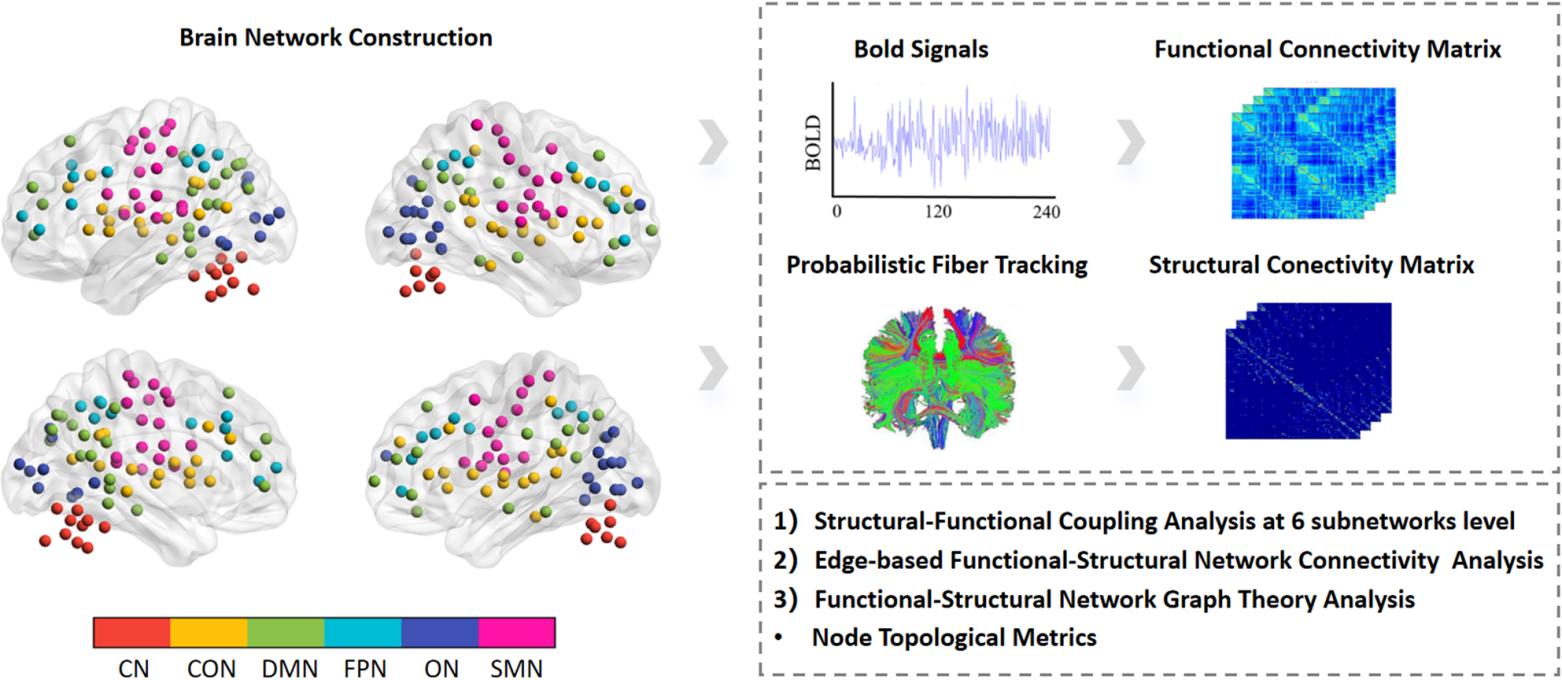
The workflow of this study. Note:* CN* cerebellum network,* CON* cingulo-opercular network,* DMN* default mode network,* FPM* fronto-parietal network,* ON* occipital network,* SMN* sensorimotor network

The structural and functional networks composed of 160 ROIs in the Dos-160 template [32]. Brain edges were defined by connectivity between brain nodes. For each node, a sphere was created with a 5 mm radius, centered on the atlas coordinates. According to the study of Dosenbach et al. [32], the 160 ROIs have been assigned into six subnetworks, including cerebellum network (CN, 18 ROIs), cingulo-opercular network (CON, 32 ROIs), default mode network (DMN, 34 ROIs), fronto-parietal network (FPN, 21 ROIs), occipital network (ON, 22ROIs) and sensorimotor network (SMN, 33 ROIs). Subsequently, further network analyses were conducted on these six subnetworks (Fig. 3).

After preprocessing, structure and function connectivity matrices were created for every individual using DPABINet V1.3 and MRtrix3 software [33]. For FC matrices, the time window was the entire scan duration. Mean regional BOLD time series were extracted for each ROI within the six subnetworks using the complete scan time series. Pearson correlation coefficients were then computed between all ROI pairs and transformed using Fisher’s z to construct FC matrices for each subnetwork. These steps were performed separately for the two groups (TI and Sham) and for the two time points (pre-stimulation and post-stimulation), yielding four FC matrices per subnetwork (TI–pre, TI–post, Sham–pre, Sham–post). Similarly, for the SC matrices, weighted, undirected connectomes were constructed using the tck2connectome command while scaling each contribution to the connectome edge by the inverse of its two node volumes [34]. This adjustment helps reduce bias caused by larger parcels having a higher probability of being intersected by any streamline [35]. This method offers the advantage of covering anatomically compact or atrophic areas that inherently have a limited grey matter–white matter interface for streamline initiation. For FC matrices, Pearson correlation coefficients were calculated between all ROI pairs using their average regional BOLD time series, followed by Fisher’s z-transformation.

### Network analysis

To investigate the effects of TI stimulation on brain functional and structural networks, we first conducted structural-functional connectivity coupling analysis on six subnetworks. This analysis involved computing Pearson’s correlation coefficients between the SC matrices and FC matrices obtained for each condition (pre-stimulation and post-stimulation) and each group (TI and Sham). These correlation coefficients, derived for each participant, served as metrics for assessing the SC-FC coupling within the subnetworks. Consistent with previous research [36], we only correlated the non-zero edges in the SC matrices with the FC matrices, and primarily focused on changes in SC-FC coupling values before and after intervention. Furthermore, we investigated the effects of TI stimulation on edge-based intra-network connectivity across six subnetworks by directly comparing differences in functional and structural network connectivity matrices both between and within groups. Finally, we calculated several common nodal topological brain network metrics for six functional and structural subnetworks using DPABINet V1.3. Regional nodal metrics included nodal efficiency, nodal degree and nodal betweenness. A sparsity threshold range of 0.05 < S < 0.29, with an interval of 0.01, selected in this study, which was checked by previous similar study [37]. The area under the curve (AUC) across all sparsities was calculated for each network metric and fed into statistical analyses to avoid biases [38, 39].

### Correlation analysis

Pearson correlation analysis was conducted between the difference in second-stage implicit learning task performance (ΔSIL) and the difference in CN SC-FC coupling (ΔSC-FC), both showing significant changes following TI stimulation, to explore the neural mechanisms underlying TI stimulation-induced enhancement of implicit learning capacity.

### Statistical analysis

All statistical analyses were performed using SPSS version 26.0. For SC-FC coupling analysis, Pearson’s correlation coefficient was calculated between nonzero edges of the SC network and corresponding elements of the FC matrix, with independent-sample t-tests comparing coupling values between groups. To control for type I error inflation resulting from multiple comparisons across six brain networks, the p-values from the independent t-tests were adjusted using the False Discovery Rate (FDR) correction. Linear Mixed-Effects Model (LMM) were employed to analyze all primary outcome measures (static and dynamic fALFF, FIL, and SIL). The fixed effects in the model included Treatment (TI vs. Sham), Period (Pre vs. Post), and Sequence (TI-Sham vs. Sham-TI), along with the Treatment × Period interaction term. Crucially, this interaction term was included to test for potential carryover effects inherent to the crossover design. Baseline performance, participant identification, sequence (TI-first vs. Sham-first) and period (four testing time points in total) were included as random effect. For the behavioral measure SIL, to rigorously control for task-related practice effects, the post-stimulation FIL score was additionally incorporated into the fixed effects as a covariate. All models were estimated using the Restricted Maximum Likelihood method. Where significant main or interaction effects were observed, post-hoc pairwise comparisons were performed with Bonferroni correction. Effect sizes are reported as Cohen’s d. All statistical analyses were conducted using SPSS 27.0, with the significance level set at α = 0.05. For the SIL analysis, the final value of FIL (post-stimulation) was entered as a covariate to control for practice-related improvements. Where significant main or interaction effects were observed, post-hoc pairwise comparisons were performed with Bonferroni correction. Questionnaire data including blinding efficacy, AEQ, and SSS were assessed with Pearson’s chi-square test, while generalized estimating equations (GEE) with Bonferroni-corrected post hoc comparisons were applied for non-normally distributed data such as MoCA scores. Statistical significance was defined as α < 0.05.

In the edge-based intra-network connectivity and nodal topology comparison, DPABI software was utilized. Specifically, two-sample t-tests were employed for between-group comparisons, while paired t-tests were conducted for within-group assessments. The FDR correction was applied to account for multiple comparisons across nodes and edges within each network, establishing a significance threshold of 0.0083 (0.05/6) to incorporate comparisons across the six networks through Bonferroni correction.

## Results

### Effects of TI stimulation on static and dynamic fALFF in target regions

**Fig. 4:**
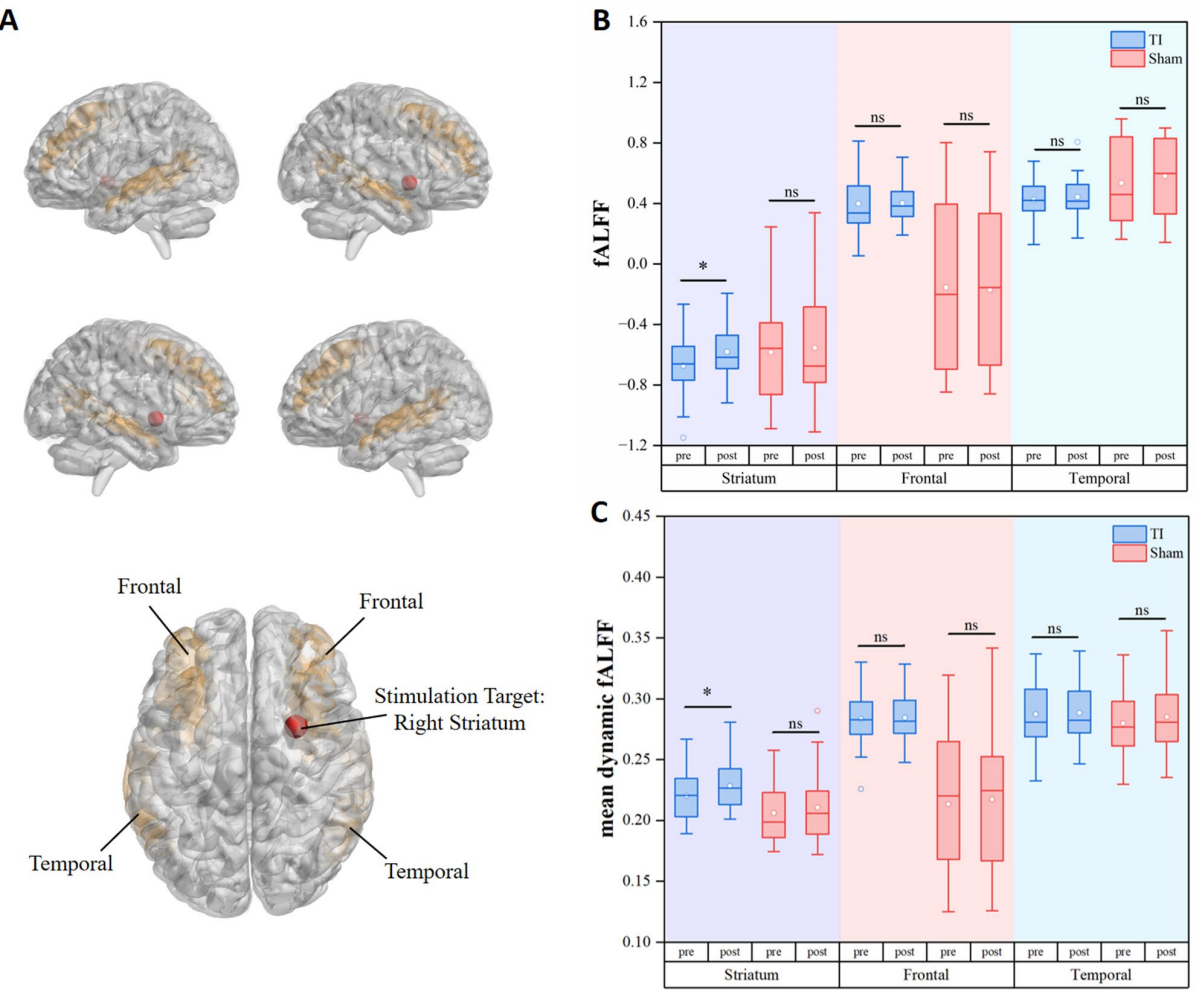
fALFF and mean dynamic fALFF activity in stimulation targets and control regions (frontal and temporal lobes) beneath electrode placement in the TI and Sham groups. Note:* TI* temporal interference stimulation,* Sham* sham stimulation,* fALFF* fractional amplitude of low-frequency fluctuations; *p < 0.05; ns, not significant (*p* > 0.05)

 Time (pre-stimulation and post-stimulation) was observed for the static fALFF. However, a significant main effect of period was observed (β = -0.094, t = – 2.549, *p* = 0.016) (Table 1). Post hoc tests showed a significant pre-to-post increase only in the TI condition (Cohen’s d = 0.49, *p* = 0.016) (Table 2; Fig. 4A). No significant differences were observed in the cortical control regions beneath electrode placement, including frontal and temporal areas (*p* > 0.05) (Tab. S1, S2).

**Table 1:** Estimated regression coefficients of the linear mixed model for fALFF and mean dynamic fALFF value of the striatum

	Estimate	SE	df	95% CI		t	*p*
				Lower	Upper		
fALFF							
Intercept	0.001	0.038	76.4	-0.075	0.077	0.030	0.978
Baseline of fALFF value	0.865	0.043	47.4	0.778	0.953	19.899	< 0.001***
Main effect of group	– 0.050	0.034	70.4	– 0.118	0.019	– 1.450	0.154
Main effect of time	– 0.092	0.036	52	– 0.164	– 0.020	– 2.547	0.016*
Inter effect: group*time	0.061	0.051	52	– 0.041	0.164	1.208	0.235
Mean dynamic fALFF							
Intercept	0.040	0.013	54.1	0.014	0.066	3.097	0.003**
Baseline of mean dynamic fALFF value	0.856	0.057	51.9	0.741	0.971	14.936	< 0.001***
Main effect of group	– 0.006	0.004	73.6	– 0.013	0.001	– 1.578	0.120
Main effect of time	– 0.008	0.004	52	– 0.016	– 0.001	– 2.179	0.035*
Inter effect: group*time	0.004	0.005	52	– 0.007	0.014	0.684	0.498

*CI* confidence interval,* df* degrees of freedom,* SE* standard error,* fALFF* fractional amplitude of low-frequency fluctuations; *, *p* < 0.05; **, *p* < 0.01; ***, *p* < 0.001

**Table 2:** Contrasts of the linear mixed model for fALFF and mean dynamic fALFF value of the striatum

Comparison	Marginal average deviation	SE	df	95% CI		Cohen’s d	*p*
				Lower	Upper		
fALFF							
TI-post vs. TI-pre	0.092	0.036	52	0.019	0.164	0.49	0.016*
Sham-post vs. Sham-pre	0.030	0.036	52	-0.042	0.102	0.09	0.406
TI-pre vs. Sham-pre	– 0.012	0.034	70.4	– 0.080	0.056	– 0.32	0.729
TI-post vs. Sham-post	0.050	0.034	70.4	– 0.019	0.118	– 0.10	0.149
Mean dynamic fALFF							
TI-post vs. TI-pre	0.008	0.004	52	0.001	0.016	0.39	< 0.035*
Sham-post vs. Sham-pre	0.004	0.004	52	– 0.003	0.012	0.17	0.233
TI-pre vs. Sham-pre	0.002	0.004	73.6	– 0.005	0.009	0.63	0.571
TI-post vs. Sham-post	0.004	0.005	52	– 0.001	0.013	0.71	0.120

* CI* confidence interval,* df* degrees of freedom,* SE* standard error; Cohen’s d, effect size, * fALFF* fractional amplitude of low-frequency fluctuations, * pre* pre-stimulation;* post* post-stimulation,* TI* temporal interference stimulation; Sham, sham stimulation; *, *p* < 0.05

The mean dynamic fALFF in the target region of the right striatum similarly showed no significant interaction effect between Group (TI and Sham) and Time (pre-stimulation and post-stimulation). However, a significant main effect of period was observed (β = -0.011, t = – 2.181, *p* = 0.035) (Table 1). Post hoc tests showed a significant pre-to-post increase only in the TI condition (Cohen’s d = 0.39, *p* = 0.035) (Table 2; Fig. 4B). No significant differences were observed in the cortical control regions beneath electrode placement, including frontal and temporal areas (*p* > 0.05) (Tab. S1, S2).

### Effects of TI stimulation on SC-FC coupling and topological properties

#### Effects of SC-FC coupling

**Fig. 5:**
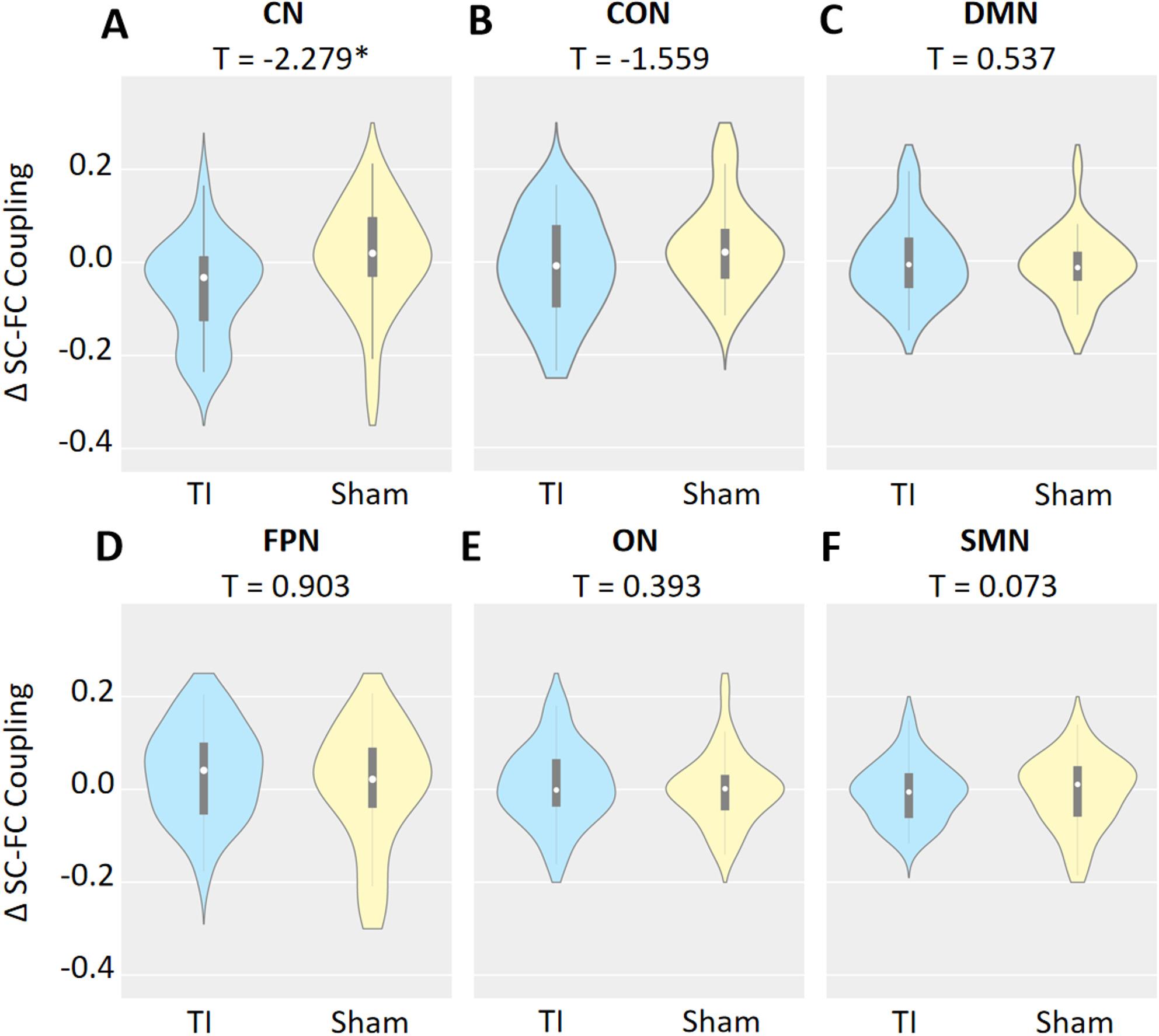
Changes in the SC-FC coupling values of brain networks in the TI and Sham groups. The violin figures show SC-FC coupling values in the:** A** Cerebellum,** B** Cingulo-Opercular,** C** Default Mode,** D** Fronto-Parietal,** E** Occipital, and** F** Sensorimotor networks. Note:* TI* temporal interference stimulation,* Sham* sham stimulation,* SC-FC coupling* structural-functional connectivity coupling; *p < 0.05 (FDR corrected)

Changes in the SC-FC coupling values of the CN network were significantly smaller in the TI group compared to the Sham group (t = – 2.279, *p* = 0.027, FDR corrected) (Fig. 5A). No significant between-group differences were observed in the CON, DMN, FPN, ON, or SMN (*p*>0.05, FDR corrected) (Fig. 5B–F).

#### Effects of edge-based intra-network connectivity

For functional networks, only within-group comparison in the TI group revealed significantly increased intra-network FC within CN (med cerebellum to lat cerebellum, med cerebellum to med cerebellum) as well as within CON (thalamus to mid insula) following stimulation (*p* < 0.05, FDR corrected) (Fig. S2A, B). However, further correction using Bonferroni adjustment for multiple comparisons across the six functional networks resulted in no significant findings overall (*p* < 0.0083, FDR corrected with Bonferroni correction).

For structural networks, no significant changes were observed in either within-group or between-group comparisons for the CN, CON, DMN, FPN, ON, or SMN.

#### Effects of network nodal topological metrics

**Fig. 6:**
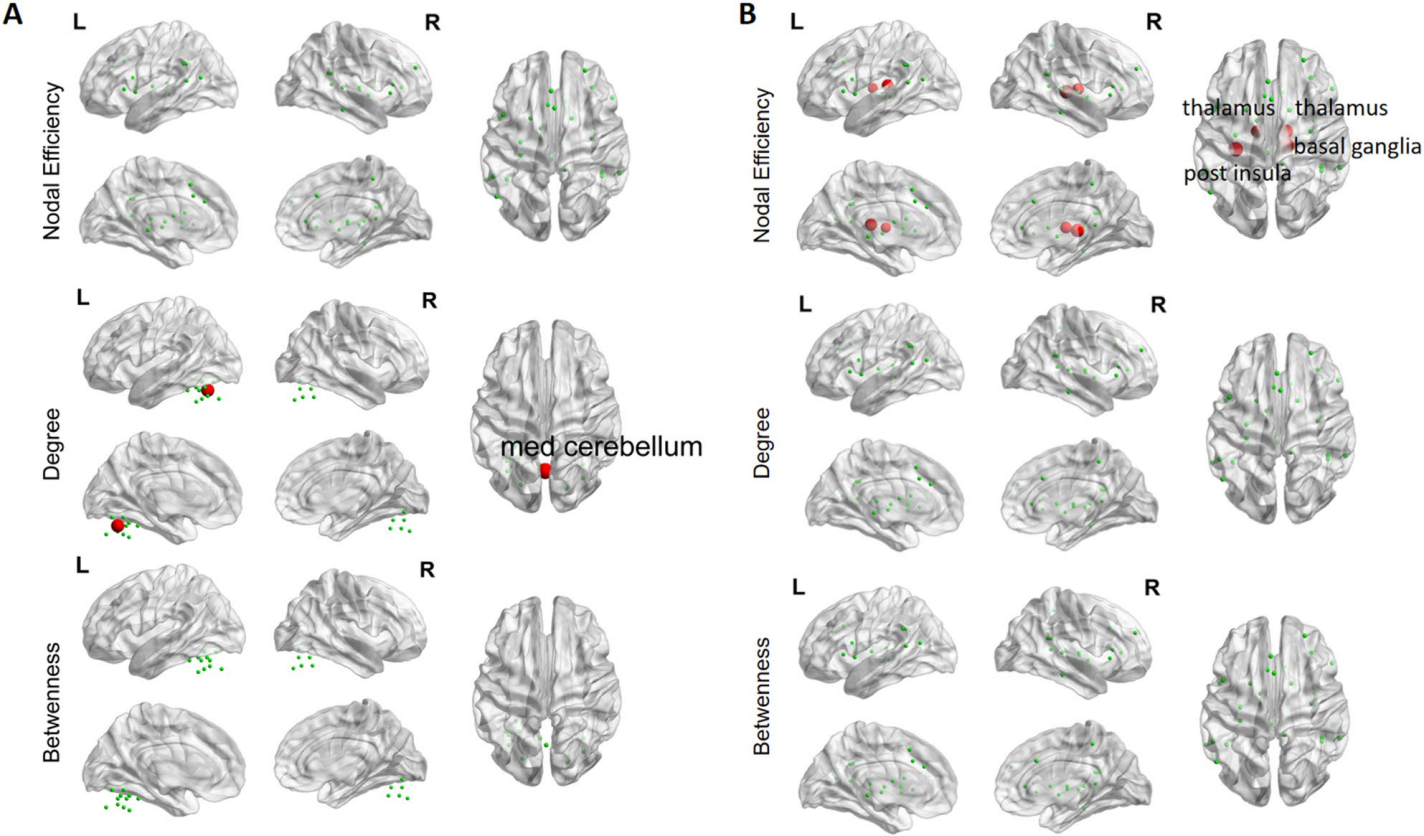
Changes in nodal topological metrics in functional networks following stimulation in TI group.** A** Cerebellar network.** B** Cingulo-opercular network. Note: Red dots indicate brain regions showing significantly increased nodal topological metrics post-stimulation compared to baseline. Results shown are corrected for multiple comparisons using the FDR correction (*p* < 0.0083), along with Bonferroni correction

For functional networks, within-group comparison in the TI group revealed significantly increased nodal degree in the CN (med cerebellum) (Fig. 6A), increased nodal efficiency in the CON (thalamus, basal ganglia and post insula) following stimulation (*p* < 0.0083, FDR corrected with Bonferroni correction) (Fig. 6B). No significant differences were found in the between-group comparison. Additionally, no significant changes were observed in either within-group or between-group comparisons for the DMN, FPN, or ON.

For structural networks, no significant changes were observed in either within-group or between-group comparisons for the CN, CON, DMN, FPN, ON, or SMN.

### Effects of TI stimulation on motor learning performance

**Fig. 7:**
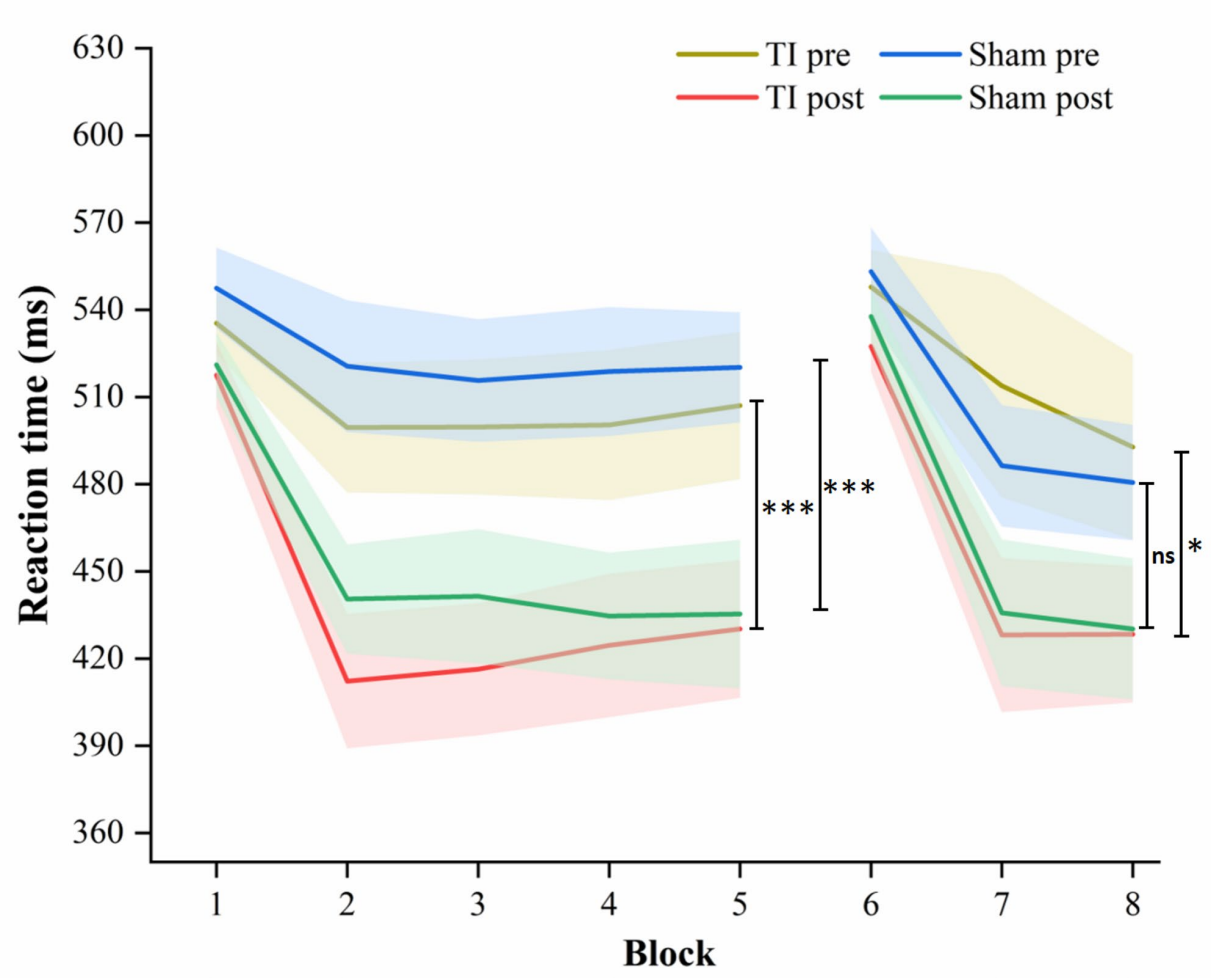
Reaction times for each block of the serial reaction time task in the TI group and Sham group. Note:* TI* temporal interference stimulation,* Sham* sham stimulation; ***p < 0.001; **p < 0.01; ns, not significant (*p* > 0.05)

For FIL, no significant interaction effect between Group (TI and Sham) and Time (pre-stimulation and post-stimulation) was observed. However, a significant main effect of period was observed (β = -62.938, t = -4.858, *p* < 0.001) (Table 3). Post hoc tests showed a significant pre-to-post increases only in the TI condition (Cohen’s d = 0.62, *p* < 0.001) and Sham group (Cohen’s d = 0.56, *p* < 0.001) (Table 4; Fig. 7). No additional pairwise comparisons yielded statistically significant differences (Table 4).

**Table 3:** Estimated regression coefficients of the linear mixed model for FIL and SIL

	Estimate	SE	df	95% CI		t	*p*
				Lower	Upper		
FIL							
Intercept	64.250	9.358	88.8	45.656	82.843	6.866	< 0.001***
Baseline of FIL value	0.960	0.054	45.0	0.851	1.069	17.677	< 0.001***
Main effect of group	– 12.067	11.846	41.6	– 35.979	11.844	– 1.019	0.318
Main effect of time	– 62.902	12.956	45.3	– 88.993	– 36.811	– 4.855	< 0.001***
Inter effect: group*time	12.150	17.142	23	– 23.310	47.610	0.709	0.489
SIL							
Intercept	43.157	12.137	83.4	19.043	67.271	3.556	0.001***
Baseline of SIL value	0.579	0.0612	47.3	0.455	0.702	9.419	< 0.001***
Final value of FIL	0.358	0.074	56.3	0.210	0.506	4.848	< 0.001***
Main effect of group	– 10.981	13.634	41.8	– 38.499	16.537	– 0.805	0.421
Main effect of time	– 36.494	17.716	50.3	– 72.072	– 0.916	– 2.060	0.043*
Inter effect: group*time	21.747	22.020	22.9	– 23.817	67.312	0.988	0.336

*CI* confidence interval,* df* degrees of freedom,* SE* standard error, * FIL* first implicit learning,* SIL* second implicit learning; *, *p* < 0.05; ***, *p* < 0.001

**Table 4:** Contrasts of the linear mixed model for FIL and SIL

Comparison	Marginal average deviation	SE	df	95% CI		Cohen’s d	*p*
				Lower	Upper		
FIL							
TI-post vs. TI-pre	62.902	12.957	45.3	36.811	88.993	0.62	< 0.001***
Sham-post vs. Sham-pre	50.752	12.957	45.3	24.661	76.843	0.56	< 0.001***
TI-pre vs. Sham-pre	– 0.083	11.846	41.6	– 23.995	23.829	– 0.02	0.997
TI-post vs. Sham-post	12.067	11.846	41.8	– 11.844	35.979	0.10	0.316
SIL							
TI-post vs. TI-pre	36.494	17.716	50.3	0.916	72.072	0.47	0.043*
Sham-post vs. Sham-pre	14.747	17.502	48.3	– 20.437	49.930	0.36	0.402
TI-pre vs. Sham-pre	– 10.766	13.535	41.2	– 38.096	16.564	– 0.25	0.433
TI-post vs. Sham-post	10.981	13.634	41.8	– 16.537	38.499	– 0.01	0.426

*CI* confidence interval,* df* degrees of freedom,* SE* standard error, Cohen’s d, effect size; FIL, first implicit learning;* SIL* second implicit learning, * pre* pre-stimulation, * post* post-stimulation;* TI* temporal interference stimulation, * Sham* sham stimulation; *, *p* < 0.05; ***, *p* < 0.001

For SIL, no significant interaction effect between Group (TI and Sham) and Time (pre-stimulation and post-stimulation) was observed. However, a significant main effect of period was observed (β = -36.451, t = – 2.067, *p* = 0.043) (Table 3). Post hoc tests showed a significant pre-to-post increase only in the TI condition (Cohen’s d = 0.47, *p* = 0.043) (Table 4; Fig. 7). No additional pairwise comparisons yielded statistically significant differences (Table 4).

### Effects of TI stimulation on brain-behavior correlations

**Fig. 8:**
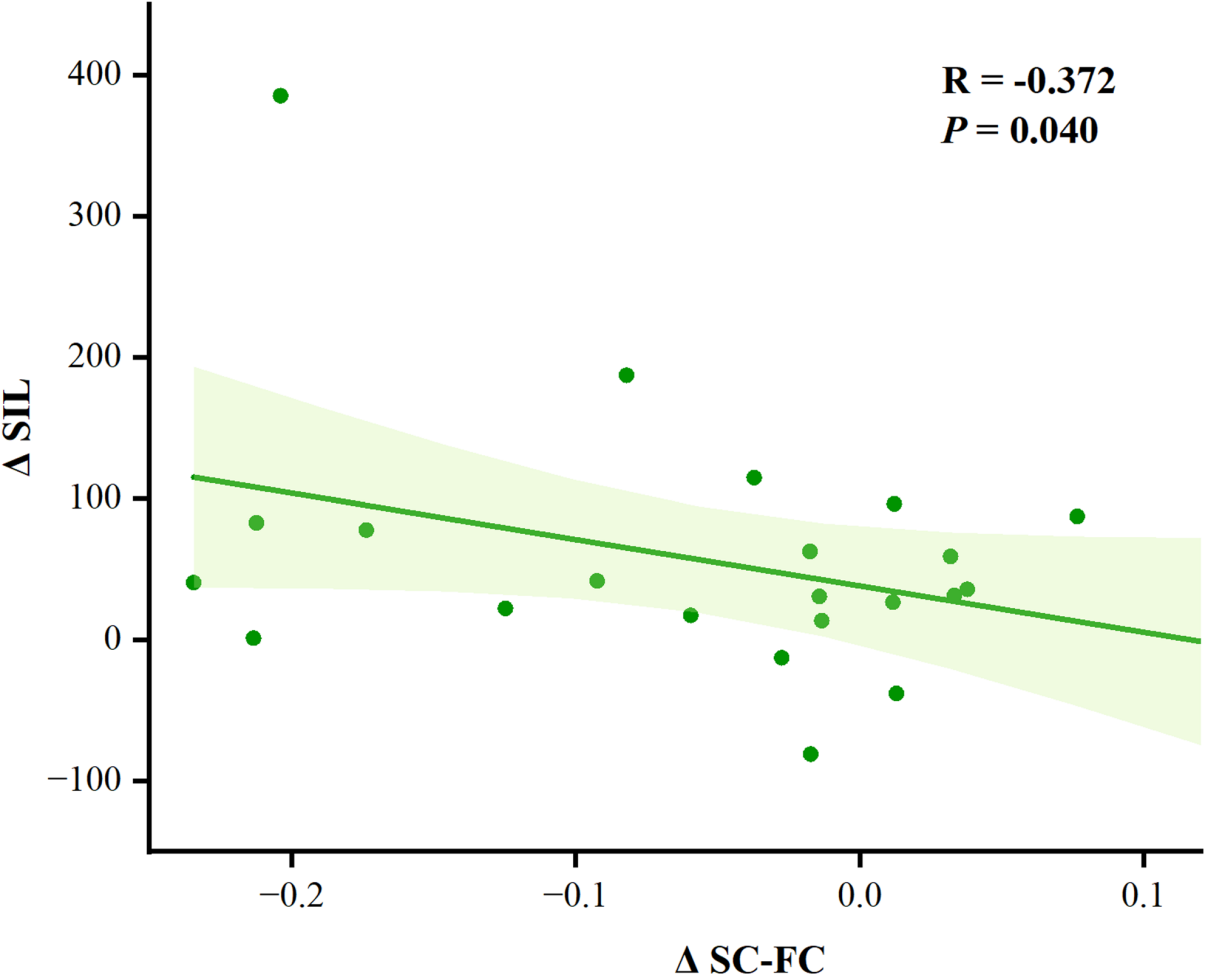
Pearson correlation analysis between changes in SIL following TI stimulation (ΔSIL) and changes in SC-FC coupling of the CN following TI stimulation (ΔSC-FC). Note:* TI* temporal interference stimulation,* SIL* second-phase implicit learning,* CN* cerebellar network,* SC-FC coupling* structural-functional connectivity coupling

Pearson correlation analysis revealed a significant negative correlation between the change in CN SC-FC coupling (ΔSC-FC) and the change in second-stage implicit learning task performance (ΔSIL) following TI stimulation (*r* = -0.372, *p* = 0.040, Fig. 8).

## Discussion

This study is the first to elucidate the mechanism by which TI stimulation of the striatum optimizes motor learning through brain network reorganization: [1] In the target region activity, TI selectively enhances static and dynamic fALFF in the deep right striatum without significant changes in cortical regions, confirming its precise targeting capability for deep nuclei; [2] In the brain network, compared to the sham group, the TI group exhibited significantly reduced SC-FC coupling in the CN. Additionally, TI stimulation significantly enhanced nodal degree in the CN and nodal efficiency in the CON, following the stimulation; [3] In the learning, the TI stimulation exhibited specific improvement during the SIL phase (*p* < 0.01); [4] In the brain-behavior correlations, the change in SC-FC coupling reduction (ΔSC-FC) in the CN showed a significant negative correlation with SIL performance improvement (ΔSIL), revealing the regulatory role of network decoupling in learning consolidation.

### Effects of TI stimulation on fALFF

This study offers the first evidence of increased resting-state activity, as measured by fALFF, in deep brain regions following TI stimulation. Following TI stimulation, both static fALFF and mean dynamic fALFF in the right striatal target region showed significant increases, while no changes were observed in the frontal/temporal cortical control regions under electrode coverage, suggesting that under post-stimulation, TI neuromodulation induced sustained after-effects primarily in the targeted deep striatal region, with no detectable after-effects in the superficial cortical control regions. However, our data cannot rule out the possibility of transient cortical activation during the TI stimulation itself, which may have a different temporal profile and subsided before our post-stimulation scans. Future studies employing simultaneous TI-fMRI recordings are essential to conclusively delineate the spatiotemporal specificity of TI. The significant fALFF enhancement in the striatal region reflects increased energy of spontaneous low-frequency oscillations (0.01–0.1 Hz) in local neural clusters, indicating improved synchronization of the stimulation target and striatum [40, 41]. Given the central role of the striatum in motor control and reward pathways, this oscillatory energy enhancement may optimize motor information processing efficiency through modulation of dopaminergic pathway activity, thereby affecting motor learning and executive functions [42, 43].

### Effects of TI stimulation on SC-FC coupling

Our findings indicate that TI stimulation targeting the right striatum significantly reduced SC-FC coupling in the CN after controlling for multiple comparisons using the FDR method. The reduction in SC-FC coupling within the CN suggests that TI stimulation promotes functional organization that is no longer strictly constrained by underlying structural connections, potentially providing greater functional flexibility for cerebellar circuits [44]. As we proposed that the SC-FC coupling in the cerebellum may be significantly influenced by the cortico-striato-thalamo-cerebellar circuit [45]. This observation aligns with established neuroscientific frameworks indicating that sensory-motor regions exhibit stronger structural-functional coupling to ensure rapid and reliable responses to external stimuli, whereas higher-level cognitive regions (particularly executive control networks) demonstrate lower structural-functional coupling to facilitate more adaptive neural processing for unpredictable complex cognitive tasks [44]. Additionally, our findings align with those reported by Missey et al., showing that TI stimulation significantly reduces inter-ictal epileptiform discharges and fast ripples in the hippocampus, thereby limiting their propagation throughout the brain [46]. This parallels our observation of enhanced fALFF in the striatum and decreased SC-FC coupling in the cerebellar network, suggesting that TI stimulation promotes a decoupling effect that enhances the functional dynamics of these circuits.

### Effects of TI stimulation on nodal topological metrics

Analysis of network nodal topological metrics revealed that TI stimulation significantly increased nodal degree in the CN (medial cerebellum) and enhanced nodal efficiency in the CON, which includes the thalamus, basal ganglia, and posterior insula within the functional networks. No alterations were observed in the DMN, FPN, or ON. No changes in nodal topological properties were detected in structural networks. These findings suggest that TI stimulation specifically modulates functional integration within cerebellar and cingulo-opercular circuits without affecting structural network architecture.

Nodes with high centrality can be categorized as network hubs [47, 48]. The observed elevations in nodal efficiency and degree centrality within the CN and CON networks indicate that TI stimulation potentiated the functional centrality of these regions, thereby facilitating enhanced information processing dynamics within their respective neural circuitry. It is noteworthy that the specific nodes exhibiting enhanced centrality metrics—medial cerebellum, thalamus, and basal ganglia (particularly the striatum)—collectively constitute critical components of the basal ganglia-cerebello-thalamo-cortical circuit. This circuit is highly integrated anatomically and functionally, playing decisive roles in motor control and cognitive processing [49, 50]. Moreover, clinical evidence has confirmed that the reduced nodal topological properties of the basal ganglia-cerebello-thalamo-cortical circuit are closely associated with motor symptoms in PD [51]. Notably, the present study reveals the TI stimulation-induced modulation of nodal topological attributes in the striatum, which may open the door to future studies evaluating the therapeutic potential of TI in the treatment of PD.

These results suggest that TI stimulation provides beneficial network modulation effects while offering the significant advantage of non-invasive neuromodulation with enhanced safety. This may position TI stimulation as a promising alternative therapeutic approach for PD and other disorders involving deep nuclear pathology [52]. Furthermore, recent studies have explored the application of multipolar temporal interference (mTI), which we believe has the potential to further optimize traditional TI stimulation methods, achieving improved stimulation precision and reduced off-target effects, thereby enhancing the efficacy of neurostimulation [53–56].

### Effects of TI stimulation on motor learning performance

Prior neuroimaging studies have consistently implicated the striatum in sequence learning and habit formation, even in the absence of overt reward [57, 58]. In this study, TI stimulation targeted striatum significantly improved the performance of SIL. However, in the FIL phase, both the TI group and the sham group achieved significant improvements following stimulation, without between group difference. This indicated that initial task training itself can yield substantial learning benefits regardless of whether TI stimulation is applied [59].

In the SIL phase, our study showed that the performance of TI group significant improved following stimulation. The significant enhancement in SIL performance following TI stimulation suggests a phase-specific modulation of learning processes. During the SIL phase, participants must retrieve and reconsolidate previously acquired sequence representations after interference, a process that places high demands on memory stabilization mechanisms. TI stimulation may have facilitated these neural processes, enhancing resistance to interference and promoting reconsolidation of motor memories.

This finding aligns with previous evidence showing that non-invasive stimulation tends to yield stronger effects when behavioral performance approaches a plateau or when consolidation mechanisms are dominantly engaged [60, 61]. Furthermore, the 20 Hz frequency adopted in the current TI protocol corresponds to β-band oscillations, which are known to support sensorimotor integration and motor memory retention [62, 63]. Collectively, these findings suggest that TI stimulation may reopen or strengthen neural plasticity windows in the later phases of implicit learning.

### Effects of TI stimulation on brain-behavior correlations

Correlation analysis revealed a significant negative correlation between changes in structure-function coupling within the cerebellar network following TI stimulation and improvements in implicit learning task performance. This finding provides robust empirical support for current theories of neural network plasticity—specifically, that decoupling helps free up neural resources and enhances behavioral performance [44, 64, 65]. As a critical hub for motor control and cognitive coordination, the altered coupling state of the cerebellar network may render information processing more flexible, thereby facilitating improvements in implicit learning ability [44, 64, 65].

Moreover, our study found that TI stimulation induces decoupling of cerebellar networks, indicating restored higher flexibility and information processing capacity in brain networks. The enhancement of implicit learning ability demonstrates that this neuro-modulatory effect translates into tangible functional improvements. Consequently, these results may suggest a potentially valuable perspective and contribute to the theoretical foundation for the clinical application of TI stimulation as a non-invasive therapeutic approach for conditions such as Parkinson’s disease, where motor learning deficits are present.

From a theoretical perspective, this result aligns with the “neural dynamic plasticity” hypothesis. Appropriate network decoupling does not signify systemic imbalance but rather grants local brain regions greater autonomy while maintaining overall collaboration, enabling adaptation to environmental and task-related uncertainties [44]. The structure-function decoupling induced by TI stimulation in the cerebellar network provides a neural foundation for individual cognitive flexibility and behavioral adaptation. It also suggests that dynamic brain-behavior regulation may stem from this self-organizing, self-regulating capacity of neural networks [66, 67].

### Limitations

This study has several limitations. Firstly, the small sample size may have limited our statistical power to detect subtle interaction effects and fully capture the complexity of individual differences in response to stimulation. Additionally, the exclusive inclusion of healthy participants constrains the generalizability of these findings to clinical populations. To address these limitations and enhance the translational relevance of this research, future studies should incorporate larger, more heterogeneous samples that include individuals with neurological conditions, particularly patients with Parkinson’s disease, to better evaluate the therapeutic potential of TI stimulation for motor learning rehabilitation. Secondly, our analysis predominantly focused on the immediate post-stimulation effects, which precluded understanding of long-term neuroplastic changes. Additionally, the lack of data collection during the stimulation phase limited our ability to assess the immediate effects of TI stimulation on cortical control regions. This oversight may hinder a comprehensive understanding of the dynamic responses in these areas, necessitating future research to incorporate longitudinal follow-ups that track cognitive function and adverse reactions over extended periods. Thirdly, the electric field modeling in this study was limited by computational constraints, providing only a simplified characterization of TI stimulation distribution. Our model did not account for individual anatomical variability or quantify critical parameters including scalp current density, tissue heating, and subject-specific TI envelope patterns. Future studies should employ more sophisticated finite element models incorporating individual neuroanatomical data to better simulate the electrophysiological and thermal effects of TI stimulation. Fourthly, the sham condition employed only brief ramping currents, failing to control for potential effects of high-frequency carrier waves. To rigorously isolate the temporal interference mechanism, future studies should include a high-frequency carrier control (matched 2000 Hz currents without beat frequency modulation) and a low-frequency control (20 Hz continuous stimulation). These additional controls would enable more definitive attribution of effects to temporal interference rather than carrier frequency exposure alone. Finally, the sole reliance on the serial reaction time paradigm limits generalizability across implicit motor learning processes. Future research should incorporate additional motor learning tasks, such as adaptation and procedural learning paradigms, to more comprehensively evaluate TI stimulation effects and enhance ecological validity.

## Conclusion

Personalized high-intensity targeted TI stimulation of the striatum enhances striatal activity and drives robust reorganization of brain networks, notably by decoupling SC-FC coupling within the CN and strengthening intra-network connectivity and nodal efficiency in both the CN and CON. These network-level modulations underlie improved implicit learning performance, highlighting the potential of TI stimulation as a precise and effective neuromodulation strategy for facilitating motor learning through deep nuclei and large-scale brain network reconfiguration.

## Supplementary Information

Below is the link to the electronic supplementary material.

Supplementary Material 1.

## Data Availability

The datasets generated during and/or analysed during the current study are available in the Mendeley Data repository, [https://data.mendeley.com/datasets/pwfddp8m76/1](%20https:/data.mendeley.com/datasets/pwfddp8m76/1).
