# Supplementary material for: Personalized high-intensity temporal interference stimulation decouples cerebellar networks to enhance implicit learning
Source: J Neuroeng Rehabil. 2026 Jan 2;23:54. doi: 10.1186/s12984-025-01865-9 (PMC12866286; doi:10.1186/s12984-025-01865-9)
Supplement: Supplementary file 1 — Supplementary Material 1. [file 12984_2025_1865_MOESM1_ESM.docx]

**Supplementary Material**

**Section A:** **Protocol for optimizing personalized TI stimulation electrode montage** (Main article - Section 2.3 continued)

This study utilized SimNIBS to segment the T1-weighted MRI images of the participants [1]. Dividing their heads into six tissue types and assigning corresponding conductivities: scalp (0.465 S/m), skull (0.01 S/m), cerebrospinal fluid (1.65 S/m), white matter (0.126 S/m), gray matter (0.276 S/m), and cavity (2.5e-14 S/m). After segmentation, we registered the 10-10 EEG electrode system onto the participants' scalp.

Next, using the Gmsh, we generated tetrahedral finite element meshes of the participants' heads. After obtaining the meshes (~3 M tetrahedral elements, 0.4 mm average edge length around the target), we solved the Laplace equation ∇·(σ∇φ)=0 using SimNIBS 4.0.1 with second-order shape functions. The PETSc-CG solver (relative tolerance 10⁻⁶, AMG preconditioner) was used under Neumann boundary conditions on electrode surfaces. Current was injected at 5 mA per channel. The resulting nodal potentials were interpolated onto a 1 mm isotropic grid in MNI space for group-level analysis. The electric field intensity was calculated separately for each of the two electrode pairs. Let E1 represent the magnitude of the electric field at each element of the grid (head model) due to a 5-mA current passing through one pair of electrodes, and E2 represent the electric field magnitude for the other pair of electrodes. The maximum amplitude modulation of the interference pattern is given by the following equation [2]:

$${TI}_{max}=\left\{ \begin{aligned} 2E_{2}, if E_{2}<E_{1}cos(a) \\ \frac{(2\left| \vec{E_{2}}\times(\vec{E_{1}}- \vec{E_{2}}) \right|)}{\left| \vec{E_{1}}- \vec{E_{2}} \right|}, &otherwise \end{aligned} \right.$$

Here, E2<E1 and a < 90 degrees, and note that the TI field amplitude is limited to the weaker of the two fields E1 and E2. From this we can get the electric field strength of each TI simulation.

Based on the electric field strength simulation calculations from prior stimulation studies targeting the striatum, the computed electric field strength was approximately 0.22 V/m [3]. Considering that this study employs a dual-channel 4-electrode stimulation paradigm with a current intensity of 5 mA for each electrode, we estimate that the electric field strength at the stimulation site will reach at least 2.2 V/m. Through simulation studies of multiple electrode placement configurations and applying the following method of exclusion, we determined the most suitable electrode placement scheme for each participant:

a) The ratio of the envelope electric field amplitude of the target brain region to the envelope electric field amplitude of the entire brain.

b) Maintain safe current densities, typically less than 2 mA/cm²

c) The magnitude of the electric field in the target area is at least 2.2 V/m

To identify the optimal four-electrode montage under the above constraints, a genetic algorithm (GA) was embedded within the individualized head-model and electric-field pipeline. The search space comprised the 81 positions of the extended 10–10 system; each chromosome encoded a unique, alphabetically sorted set of four electrode labels corresponding to the two stimulation channels. The fitness function invoked the previously validated TI solver to compute the peak electric field within a 10-mm-radius ROI centered at Montreal Neurological Institute (MNI) coordinates (28, 4, -4) in the striatum, while simultaneously enforcing the safety requirement that current density remain below 2 mA/cm²; non-compliant individuals received zero fitness. GA parameters were population size 50, crossover probability 0.8, mutation probability 0.1, elite retention 10, and termination after 200 generations or when the field strength reached 2.2 V/m. Selection combined roulette-wheel sampling with elitism; crossover employed single-point exchange followed by immediate deduplication and reordering; mutation replaced a single electrode at random. The algorithm returned the montage that maximized the focal electric field while satisfying all safety and selectivity criteria.

Through the above method screening, we obtained the optimal electrode placement position for each subject (Fig S1).


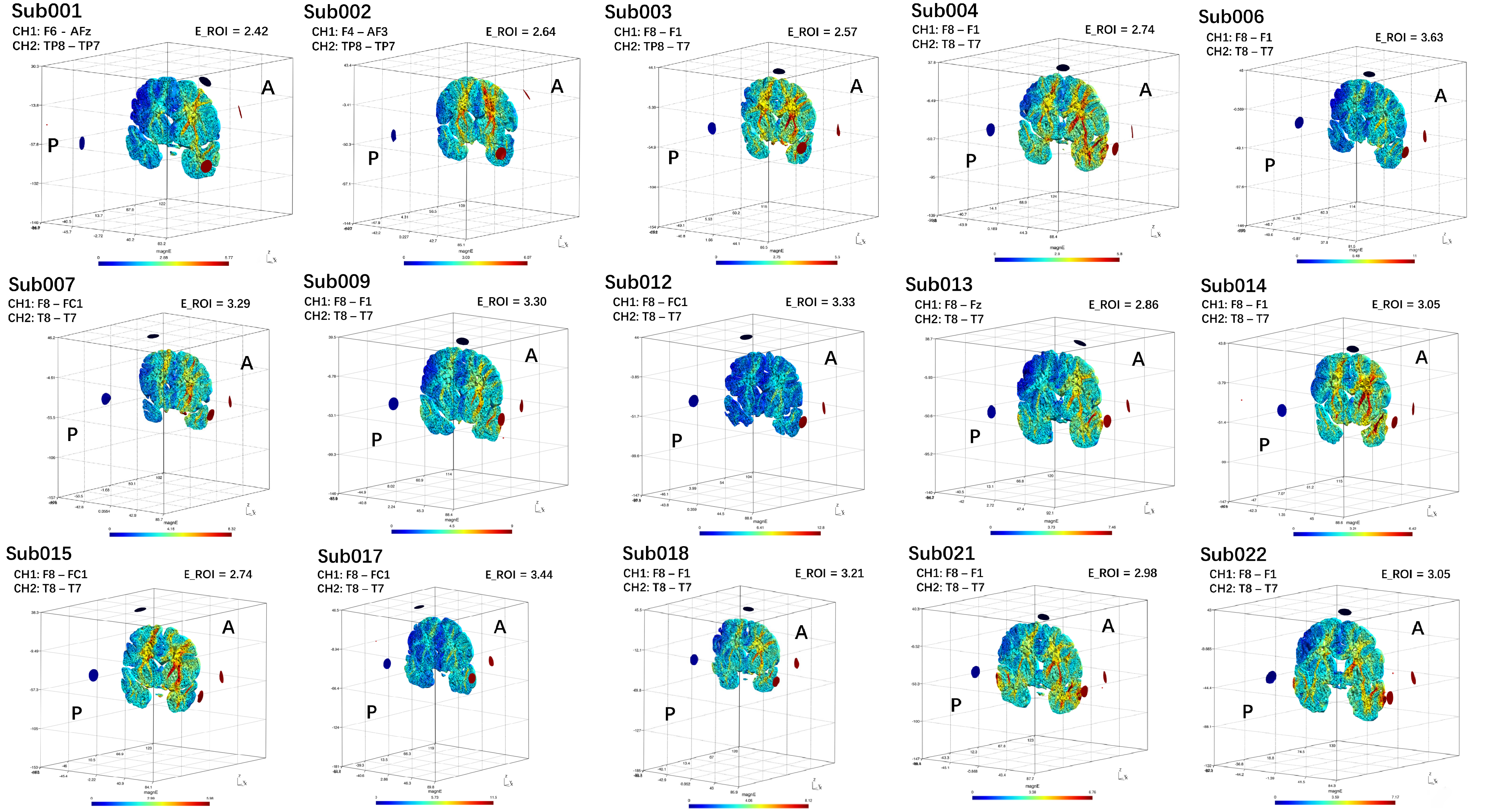


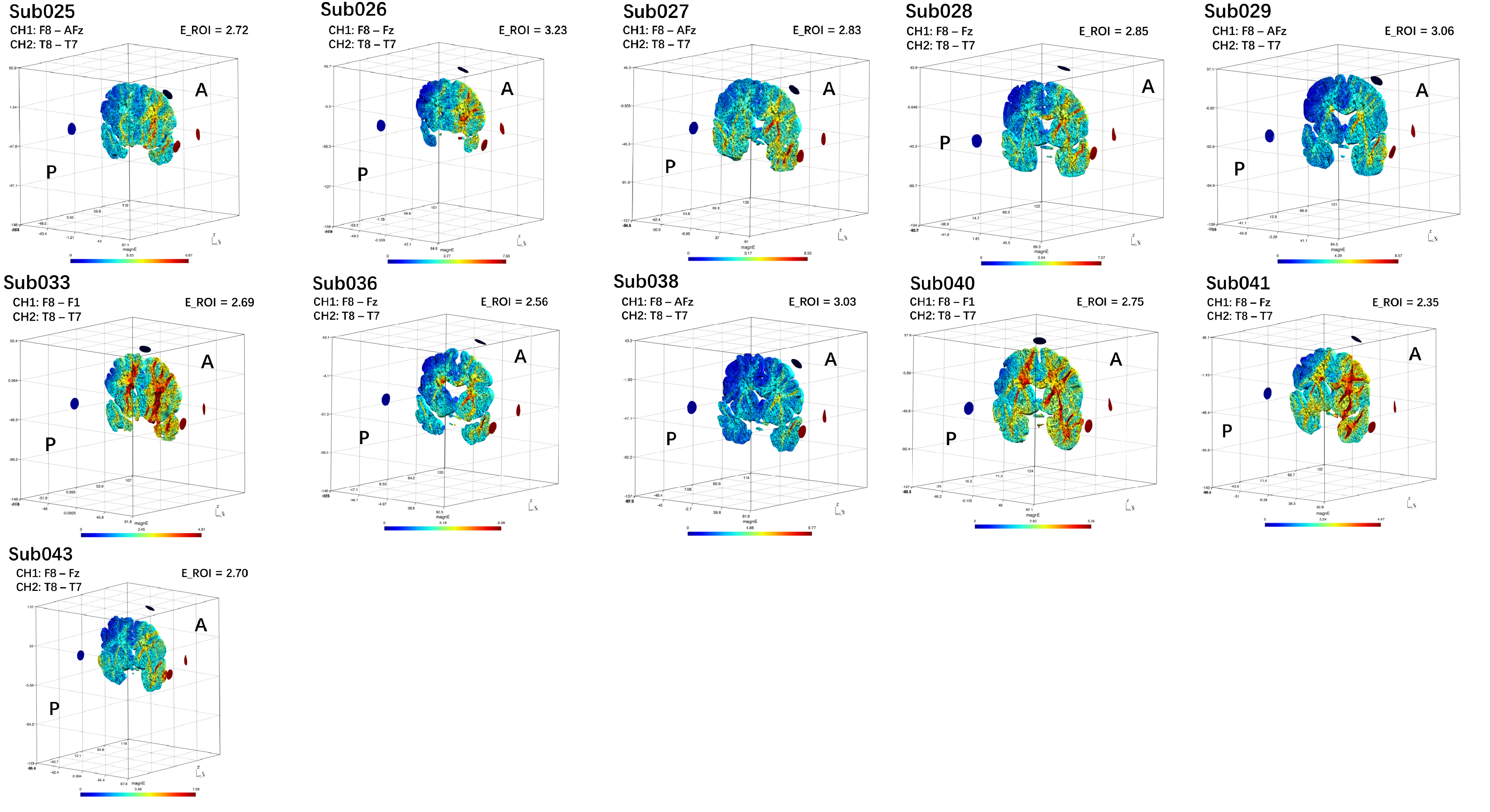


**Fig. S1. Electric field intensity for participants.** The modeling process included brain tissue segmentation, electrode placement (10-10 EEG system), finite element meshing, and TI electric field solution. A, Anterior; P, Posterior; E_ROI, Electric field intensity in the ROI area.

**Section B: Extended analysis of main results**

| **Tab.S1. Estimated regression coefficients of the linear mixed model for fALFF and mean dynamic fALFF value of the temporal** | | | | | | | |
| --- | --- | --- | --- | --- | --- | --- | --- |
|  | Estimate | SE | df | 95% CI | | t | *p* |
|  |  |  |  | Lower | Upper |  |  |
| fALFF | | | | | | | |
| Intercept | 0.080 | 0.022 | 91.7 | 0.037 | 0.123 | 3.659 | < 0.001^***^ |
| Baseline of fALFF value | 0.855 | 0.034 | 52.0 | 0.786 | 0.924 | 24.835 | < 0.001^***^ |
| Main effect of group | 0.045 | 0.023 | 102.6 | -0.001 | 0.091 | 1.930 | 0.056 |
| Main effect of time | -0.018 | 0.025 | 52.0 | -0.068 | 0.031 | -0.749 | 0.457 |
| Inter effect: group*time | -0.029 | 0.035 | 52.0 | -0.099 | 0.041 | -0.832 | 0.409 |
| Mean dynamic fALFF | | | | | | | |
| Intercept | 0.073 | 0.017 | 44.0 | 0.039 | 0.107 | 4.325 | < 0.001^***^ |
| Baseline of mean dynamic fALFF value | 0.750 | 0.058 | 41.9 | 0.634 | 0.866 | 13.040 | < 0.001^***^ |
| Main effect of group | 0.002 | 0.004 | 50.2 | -0.006 | 0.011 | 0.522 | 0.604 |
| Main effect of time | -0.001 | 0.005 | 53.1 | -0.011 | 0.009 | -0.211 | 0.834 |
| Inter effect: group*time | -0.004 | 0.007 | 28.9 | -0.018 | 0.009 | -0.629 | 0.534 |
| Note: CI, confidence interval; df, degrees of freedom; SE, standard error; fALFF, fractional amplitude of low-frequency fluctuations; ^***^, *p*< 0.001. | | | | | | | |

| **Tab. S2.** **Contrasts of the linear mixed model for fALFF and mean dynamic fALFF value of the temporal** | | | | | | | |
| --- | --- | --- | --- | --- | --- | --- | --- |
| Comparison | Marginal average deviation | SE | df | 95% CI | | Cohen's d | *p* |
|  |  |  |  | Lower | Upper |  |  |
| fALFF | | | | | | | |
| TI-post vs. TI-pre | 0.018 | 0.025 | 52.0 | -0.031 | 0.068 | 0.14 | 0.457 |
| Sham-post vs. Sham-pre | 0.047 | 0.025 | 52.0 | -0.002 | 0.097 | 0.18 | 0.060 |
| TI-pre vs. Sham-pre | -0.016 | 0.023 | 102.6 | -0.062 | 0.030 | -0.50 | 0.493 |
| TI-post vs. Sham-post | -0.045 | 0.023 | 102.6 | -0.091 | 0.001 | -0.72 | 0.056 |
| Mean dynamic fALFF | | | | | | | |
| TI-post vs. TI-pre | 0.001 | 0.005 | 53.1 | -0.009 | 0.011 | 0.04 | 0.834 |
| Sham-post vs. Sham-pre | 0.005 | 0.005 | 53.1 | -0.005 | 0.015 | 0.18 | 0.303 |
| TI-pre vs. Sham-pre | 0.002 | 0.004 | 50.2 | -0.007 | 0.011 | 0.36 | 0.667 |
| TI-post vs. Sham-post | -0.002 | 0.004 | 50.2 | -0.011 | 0.006 | 0.13 | 0.604 |
| Note: CI, confidence interval; df, degrees of freedom; SE, standard error; Cohen's d, effect size; fALFF, fractional amplitude of low-frequency fluctuations; pre, pre-stimulation; post, post-stimulation; TI, temporal interference stimulation; Sham, sham stimulation. | | | | | | | |

| **Tab.S3. Estimated regression coefficients of the linear mixed model for fALFF and mean dynamic fALFF value of the frontal** | | | | | | | |
| --- | --- | --- | --- | --- | --- | --- | --- |
|  | Estimate | SE | df | 95% CI | | t | *p* |
|  |  |  |  | Lower | Upper |  |  |
| fALFF | | | | | | | |
| Intercept | 0.023 | 0.018 | 102.3 | -0.012 | 0.058 | 1.325 | 0.188 |
| Baseline of fALFF value | 0.950 | 0.019 | 52.0 | 0.913 | 0.988 | 50.851 | < 0.001^***^ |
| Main effect of group | -0.045 | 0.025 | 102.5 | -0.094 | 0.004 | -1.832 | 0.070 |
| Main effect of time | -0.003 | 0.023 | 52.0 | -0.050 | 0.043 | -0.145 | 0.885 |
| Inter effect: group*time | 0.018 | 0.033 | 52.0 | -0.048 | 0.083 | 0.541 | 0.591 |
| Mean dynamic fALFF | | | | | | | |
| Intercept | 0.005 | 0.006 | 52.3 | -0.007 | 0.016 | 0.793 | 0.432 |
| Baseline of mean dynamic fALFF value | 0.988 | 0.020 | 47.3 | 0.949 | 1.027 | 50.475 | < 0.001^***^ |
| Main effect of group | 0.002 | 0.003 | 57.3 | -0.003 | 0.007 | 0.716 | 0.477 |
| Main effect of time | -0.001 | 0.002 | 51.0 | -0.006 | 0.004 | -0.490 | 0.626 |
| Inter effect: group*time | -0.003 | 0.003 | 26.0 | -0.009 | 0.004 | -0.852 | 0.402 |
| Note: CI, confidence interval; df, degrees of freedom; SE, standard error; fALFF, fractional amplitude of low-frequency fluctuations; ^***^, *p*< 0.001. | | | | | | | |

| **Tab. S4.** **Contrasts of the linear mixed model for fALFF and mean dynamic fALFF value of the frontal** | | | | | | | |
| --- | --- | --- | --- | --- | --- | --- | --- |
| Comparison | Marginal average deviation | SE | df | 95% CI | | Cohen's d | *p* |
|  |  |  |  | Lower | Upper |  |  |
| fALFF | | | | | | | |
| TI-post vs. TI-pre | 0.003 | 0.023 | 52.0 | -0.043 | 0.050 | 0.02 | 0.885 |
| Sham-post vs. Sham-pre | -0.014 | 0.023 | 52.0 | -0.061 | 0.032 | -0.03 | 0.539 |
| TI-pre vs. Sham-pre | 0.028 | 0.025 | 102.5 | -0.021 | 0.077 | 1.32 | 0.267 |
| TI-post vs. Sham-post | 0.045 | 0.025 | 102.5 | -0.004 | 0.094 | 1.45 | 0.070 |
| Mean dynamic fALFF | | | | | | | |
| TI-post vs. TI-pre | 0.001 | 0.002 | 51.0 | -0.004 | 0.006 | 0.05 | 0.626 |
| Sham-post vs. Sham-pre | 0.004 | 0.002 | 51.0 | -0.001 | 0.009 | 0.07 | 0.114 |
| TI-pre vs. Sham-pre | 0.001 | 0.003 | 57.3 | -0.004 | 0.006 | 1.61 | 0.747 |
| TI-post vs. Sham-post | -0.002 | 0.003 | 57.3 | -0.007 | 0.003 | 1.49 | 0.477 |
| Note: CI, confidence interval; df, degrees of freedom; SE, standard error; Cohen's d, effect size; fALFF, fractional amplitude of low-frequency fluctuations; pre, pre-stimulation; post, post-stimulation; TI, temporal interference stimulation; Sham, sham stimulation. | | | | | | | |


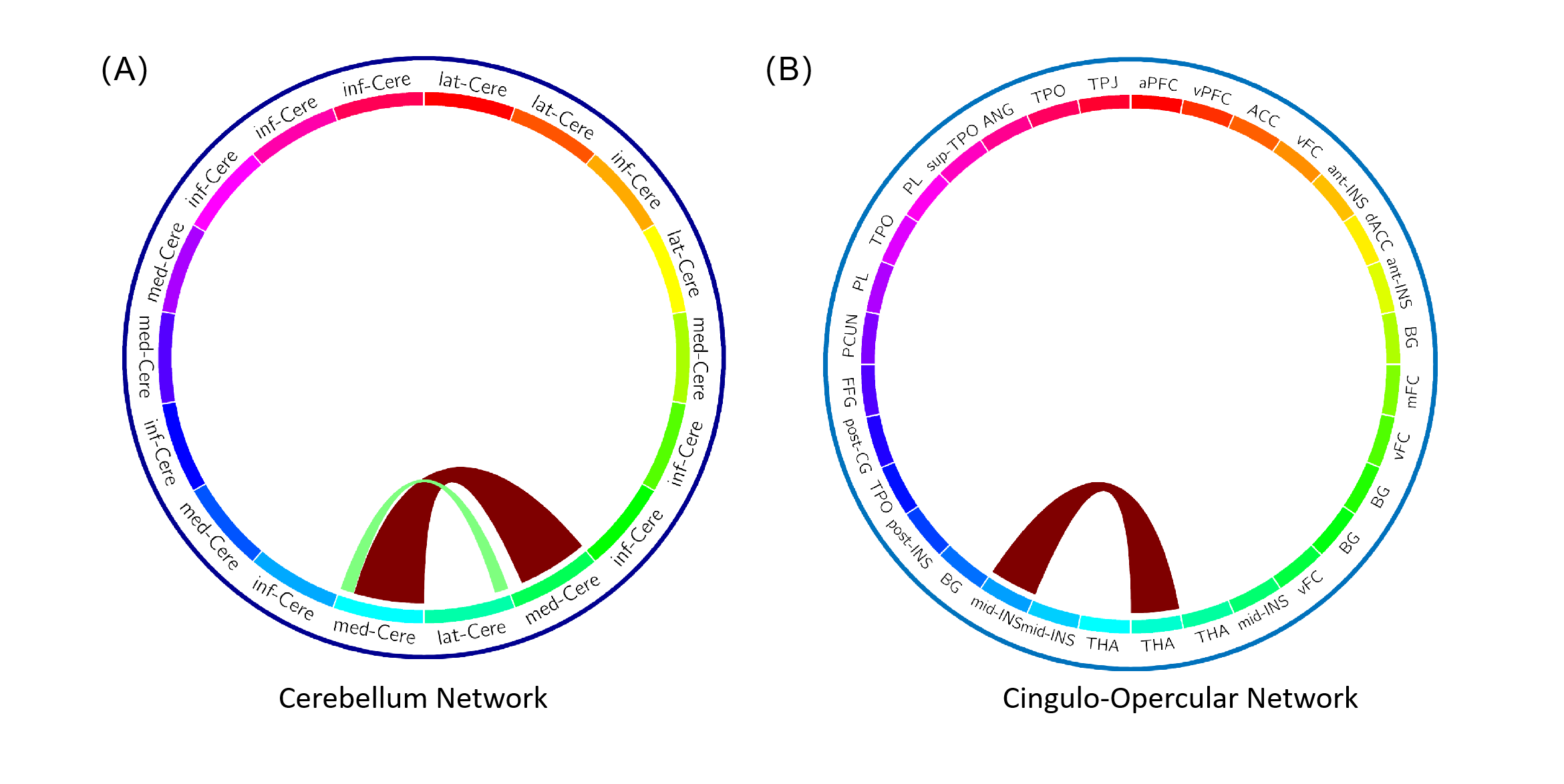


**Fig. S2. Edge-based intra-network connectivity changes following stimulation in the TI group.** (A) Cerebellar network. (B) Cingulo-opercular network. Note: med-Cere, med cerebellum; lat-Cere, lat cerebellum; THA, thalamus; mid-INS, mid insula. Results shown are corrected only with FDR (p < 0.05, FDR corrected) and are uncorrected for multiple comparisons using Bonferroni adjustment.

**
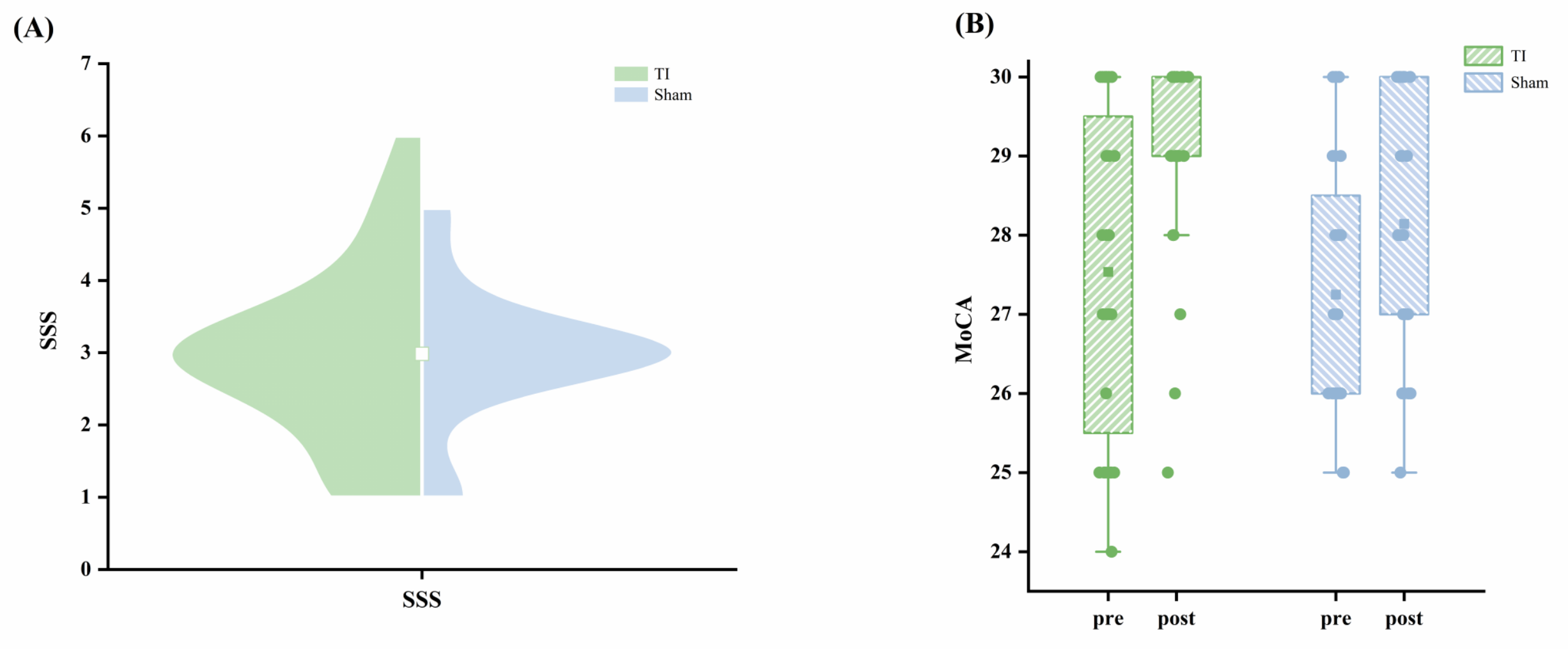
Fig. S3. Questionnaire responses about sleepiness and cognitive**

Subjects were required to complete the questionnaire before and after the experiment to account for subject wakefulness and cognitive status (n=28). **(A)** Subjects’ level of attention and fatigue before the experiment, quantified with the Stanford Sleepiness Scale, ranging from 0 to 8. Comparison of pre-stimulation sleepiness in the TI and Sham groups. No significant difference was found (Pearson's chi-square test: χ^2^ = 4.275, P = 0.513). **(B)** Both groups of subjects completed the Montreal Cognitive Assessment Scale before and after the experiment. The results of the questionnaire data were compared for differences by Generalized Estimating Equations (GEE). The results showed that there was no significant difference between the two groups on the time x group interaction effect (Wald χ^2^ = 1.295, P = 0.255), and the group main effects analysis did not reveal differences (Wald χ^2^ = 2.655, P = 0.103). Time main effects analysis showed that post-experiment cognition was significantly higher than pre-experiment (Wald χ^2^ = 22.040, P < 0.001). TI, temporal interference stimulation; Sham, sham stimulation; Pre, before the experiment; Post, after the experiment; SSS, Stanford Sleepiness Scale; MoCA, Montreal Cognitive Assessment Scale. Boxes show median and interquartile range (IQR); whiskers extend to data within 1.5×IQR; points beyond are outliers.

**
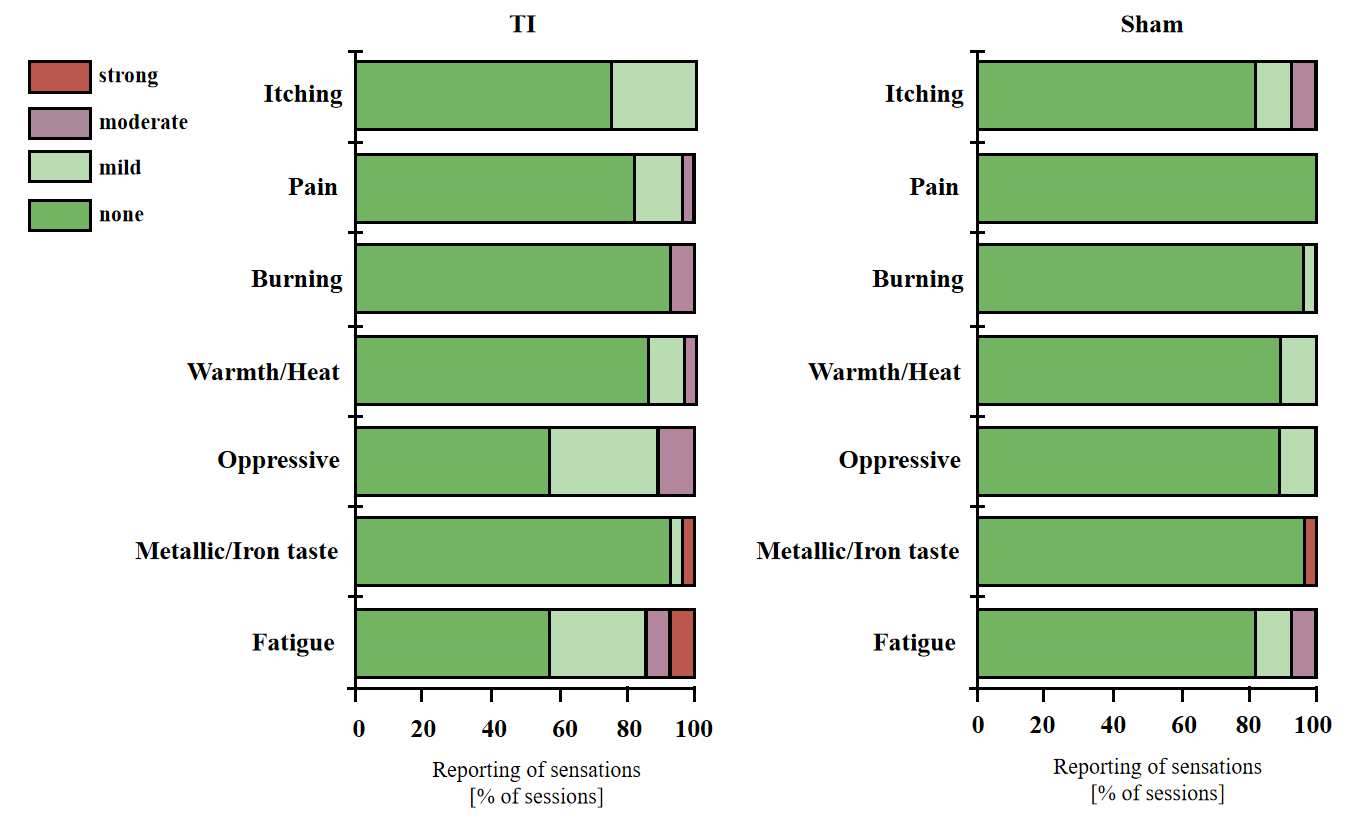
**

**Fig. S4.** **Reported side effects of TI stimulation and sham stimulation**

Subjects from both groups were asked to complete the Adverse Effects Questionnaires at the end of the experiment. The scale categorizes adverse reactions into 4 scales. The results of the questionnaire were analyzed using Pearson's chi-square test to determine the variability in the scores of the items between the two groups. The results showed that there was no significant difference between the two groups in terms of the total score of adverse reactions (χ^2^ = 10.864, P = 0.285). TI, temporal interference stimulation; Sham, sham stimulation.


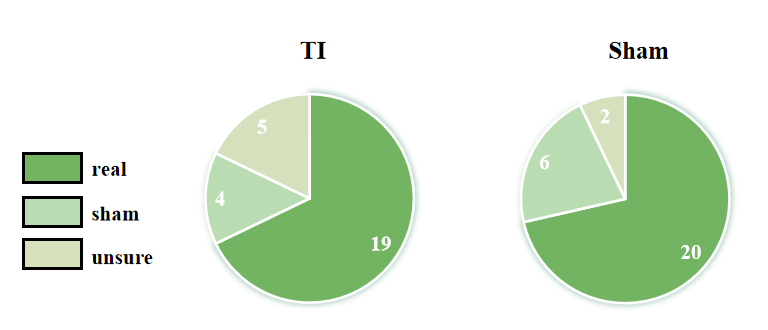
**Fig. S5. Blinding Efficacy of TI and Sham Stimulation.**

At the end of the experiment, subjects were asked to complete a blind school test questionnaire. The results of the questionnaire were tested by Pearson's chi-square and showed that without difference in the blinded test of stimuli across groups (χ2 = 1.711, P = 0.425).

**Reference**

[1] Thielscher A, Antunes A, Saturnino GB. Field modeling for transcranial magnetic stimulation: A useful tool to understand the physiological effects of TMS? Annu Int Conf IEEE Eng Med Biol Soc 2015;2015:222-5.

[2] Grossman N, Bono D, Dedic N, Kodandaramaiah SB, Rudenko A, Suk HJ, et al. Noninvasive Deep Brain Stimulation via Temporally Interfering Electric Fields. Cell 2017;169(6):1029-41.e16.

[3] Wessel MJ, Beanato E, Popa T, Windel F, Vassiliadis P, Menoud P, et al. Noninvasive theta-burst stimulation of the human striatum enhances striatal activity and motor skill learning. Nat Neurosci 2023;26(11):2005-16.
